# A reduction in the vascular smooth muscle cell focal adhesion component syndecan‐4 is associated with abdominal aortic aneurysm formation

**DOI:** 10.1002/ctm2.605

**Published:** 2021-12-22

**Authors:** Jiaxin Hu, Yuyu Li, Zhonghai Wei, Haiting Chen, Xuan Sun, Qing Zhou, Qi Zhang, Yong Yin, Meng Guo, Jianzhou Chen, Guangyao Zhai, Biao Xu, Jun Xie

**Affiliations:** ^1^ Department of Cardiology, Nanjing Drum Tower Hospital The Affiliated Hospital of Nanjing University Medical School, MOE Key Laboratory of Model Animal for Disease Study, Nanjing University Nanjing China; ^2^ Department of Cardiac Surgery, Nanjing Drum Tower Hospital The Affiliated Hospital of Nanjing University Medical School Nanjing University Nanjing China; ^3^ Department of Cardiology, Beijing Anzhen Hospital Capital Medical University Beijing China

**Keywords:** abdominal aortic aneurysm, F/G‐actin‐MRTF‐A, phenotypic change, RhoA, syndecan‐4

## Abstract

**Background:**

Abdominal aortic aneurysm (AAA) is a serious vascular disease for which there is no effective drug treatment. The incidence of AAA increases significantly as a subject ages, and the molecular mechanism of AAA formation remains elusive. In the present study, we investigated the role of syndecan‐4 (SDC4), an important component of focal adhesions, in AAA formation and its association with phenotypic changes in vascular smooth muscle cells (VSMCs).

**Methods and results:**

The protein expression levels of SDC4 were significantly decreased in human AAA tissue and those of an AAA mouse model. Moreover, SDC4 knockout (KO) in mice accelerated the formation and rupture of AAAs induced by angiotensin II (Ang II) and calcium chloride (CaCl_2_)

Mechanistically, the decrease in SDC4 led to the transformation of cultured VSMCs from a contractile to a secretory phenotype. The RhoA‐F/G‐actin‐myocardin‐related transcription factor‐A (MRTF‐A) signalling pathway was shown to be involved in SDC4‐dependent VSMC alteration. Sphingosine‐1‐phosphate (S1P), a G‐protein‐coupled receptor, attenuated the AAA formation in SDC4‐KO and wild‐type (WT) mice in response to Ang II and CaCl_2_ stimulation.

**Conclusion:**

We herein demonstrated that silencing SDC4 was associated with increased AAA formation and phenotypic changes in VSMCs via the RhoA‐F/G‐actin‐MRTF‐A pathway. These findings indicated that a reduction in SDC4 expression was an important pathological alteration and potential therapeutic target for AAA formation.

## Graphical abstract

1. Downregulation of SDC4 was observed in human AAA sample and AAA animal model.

2. Loss of SDC4 promoted VSMC contractile phenotype alternation via RhoA‐F/G‐actin‐MRTFA pathway, which ultimately led to the formation of AAA.

3. CYM‐5478, a RhoA agonist, was used to successfully prevent AAA formation in the SDC4 KO and WT mice.

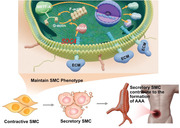

**Abbreviations**
AAA: abdominal aortic aneurysm
Ang II: angiotensin II
CaCl_2_
: calcium chloride
DHE: dihydroethidium
EVG: elastica van Gieson
GPCR: G protein‐coupled receptor
MRTF‐A: myocardin‐related transcription factor‐A
ROS: reactive oxygen species
S1P: sphingosine‐1‐phosphate
SDC1: syndecan‐1
SDC2: syndecan‐2
SDC3: syndecan‐3
SDC4: syndecan‐4
SDC4‐KD: syndecan‐4 knockdown
SDC4‐KO: syndecan‐4 knockout
SDC4‐OE: syndecan‐4 overexpression
TAA: thoracic aortic aneurysm
VSMCs: vascular smooth muscle cells； IL‐6,interleukin‐6; ELISA, enzyme linked immunosorbent assay

## 1 INTRODUCTION

Aortic aneurysms are defined as those with a diameter ≥3 cm or a local aortic diameter greater than 150% of the normal diameter.^1^ Epidemiological evidence shows that the incidence rate in men over 50 years old is 3%, of which 2.1% of men over 65 years old die from aneurysm rupture.^2^ According to the location, aortic aneurysms can be classified as thoracic aortic aneurysms (TAAs) or abdominal aortic aneurysms (AAAs). The incidence of AAA is approximately nine times higher than that of TAAs.^3^ Without timely treatment, the aortic wall remains weak and is unable to sustain intraluminal blood pressure, leading to progressive expansion and rupture. However, the current mainstay of AAA treatment is surgical repair.^4^ Due to the lack of an effective agent to treat the cause of AAA, patients still face aortic enlargement and aneurysm recurrence even after surgery, which leads to the failure of surgical treatment and seriously affects patient prognosis. Therefore, the development of new treatment options is clinically important for treating AAA. Numerous studies have reported that the mechanism of AAA formation is characterized by vascular matrix remodelling, chronic inflammation and changes in the vascular smooth muscle cell (VSMC) phenotype,^5^, ^6^ but the specific pathogenesis is still unknown. Obtaining deeper insight into the mechanisms underlying AAA development and progression is essential for identifying novel therapeutic targets.

Syndecan‐4 (SDC4), an important component of focal adhesions, plays an important role in mechanotransduction and regulates many cellular processes through signalling pathways that influence cell migration, proliferation, and endocytosis.^7^, ^8^ Previous studies by our group have indicated that SDC4 was downregulated in conditions of chronic inflammation, leading to impaired vascular homeostasis.^9^ It has been reported that focal adhesions also have important physiological significance for VSMCs.^10^ VSMCs enable intracellular mechanical signalling by coupling focal adhesions with elastin in the extracellular matrix, which regulates intracellular cytoskeletal rearrangement and maintains a contractile cellular phenotype.^11^ Therefore, the objective of this study was to explore the role of SDC4 in the occurrence and development of AAA.

We measured the levels of SDC4 in tissue from a mouse model and patients with AAA and found significantly decreased expression in AAA tissues. Furthermore, we discovered that SDC4 knockdown promoted VSMC transformation into secretory phenotypes and contributed to AAA formation in the mouse model.

## 2 METHODS

The expanded Materials and Methods section can be found in the Supplementary Materials.

### 2.1 Human aortic samples

All protocols for the use of human aortic specimens were approved by the Ethics Committee of the School of Medicine, Nanjing University (number: 2019‐190‐01). The investigation conformed to the principles outlined in the Declaration of Helsinki, and all subjects in the study provided written consent. We acquired human AAA tissues from three patients who underwent open operative repair. The preoperative ultrasonographic diagnosis was AAA. A control sample was collected from the adjacent nonaneurysmal aorta (NA) segment of the same patient. All samples were immediately stored in a −80°C refrigerator. The levels of SDC4 and paxillin on the VSMC surface of AAA tissues were analyzed by western blotting and immunofluorescence.

### 2.2 Animal experimental protocol

All animal experimental procedures were approved by the Institutional Ethics Committee of Nanjing Drum Tower Hospital (2019AE01062) and followed the guidelines outlined in the Guide for the Care and Use of Laboratory Animals (8th edition) published by the National Institutes of Health. Apoe‐/‐ mice and C57BL/6 mice were purchased from the Model Animal Research Centre of Nanjing University (8 weeks old). SDC4‐/‐ mice with a C57BL/6 background were purchased from Jackson Laboratory. Apoe‐/‐ mice and SDC4‐/‐ mice were crossbred to obtain SDC4‐/‐apoe‐/‐ mice. The control group was age‐matched C57BL/6 mice. We provided standard feed, free water and food to the animals and kept them in an environment with controlled temperature (22 ± 1°C) and humidity (65%–70%) and a 12‐h light/dark cycle. At the end of the study, all animals were anaesthetized by isoflurane inhalation (1.5%–2%) and euthanized by cervical dislocation.

### 2.3 Aneurysm quantification

The mice were anaesthetized by isoflurane inhalation (1.5%–2%), and a midline incision was made in the abdomen to separate the surrounding tissue, exposing the aorta and heart. Saline (50 ml) was infused in the left ventricle and was drained through a right atrial incision. The tissue around the tunica adventitia was dissected under an anatomical microscope, and then the aorta was photographed. Image‐Pro Plus software was used to measure the maximum diameter of the infrarenal abdominal aortic segment. The maximal abdominal aortic diameter is defined as the outer‐to‐outer diameter of the aorta, measured from one side of the aortic wall's outer layer to the other side.^12^ Necropsy was performed on all mice that died during the experimental treatment. The abdominal aortic rupture was defined as the observation of a blood clot in the retroperitoneal cavity.^13^ AAA diameter measurements were made by researchers who were blinded to the experimental design. Another investigator was invited to determine the maximum diameter of the abdominal aorta in each mouse under different treatment conditions.

### 2.4 Statistical analysis

For all statistical tests, all values are expressed as the mean ± standard deviation (SD) or median with an appropriate interquartile range, and all tests were two‐tailed. GraphPad Prism (version 8.02) software was used for statistical analyses. One‐way ANOVA and Student's *t*‐test were used for comparisons between multiple groups and two groups, respectively. Two‐way ANOVA was used in all figures containing two genotypes and two treatments. The D'Agostino–Pearson omnibus normality test or Shapiro–Wilk normality test was used to determine whether the data satisfied the condition of normal distribution. *p*<0.05 was considered significant (*p < 0.05, **p < 0.01, ***p < 0.001, ****p < 0.0001, ns = not significant).

## 3 RESULTS

### 3.1 Reduced SDC4 expression in human and murine AAA tissues

**FIGURE 1:**
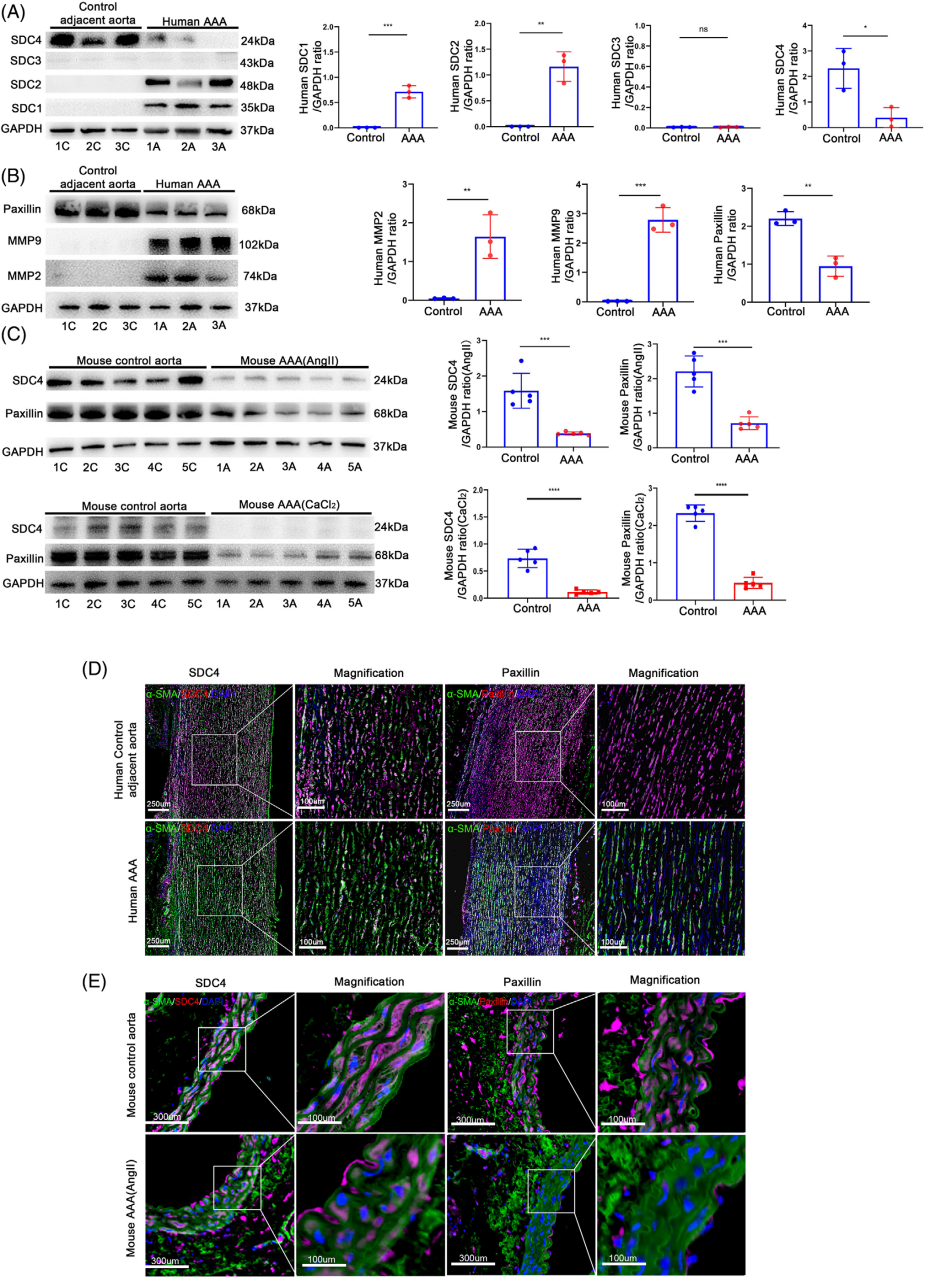
SDC4 was reduced in human and mouse abdominal aortic aneurysm (AAA) tissues. (A and B) Representative western blots of SDC1–4 (A), paxillin, MMP2 and MMP9 (B) and densitometric analysis of human AAA and adjacent control tissues (*n* = 3). (C) Representative western blots of SDC4 and paxillin and densitometric analysis of abdominal aortas from AngII‐induced AAA mice, CaCl_2_‐induced AAA mice and control mice (*n* = 5). (D) Representative immunofluorescence images of SDC4 and paxillin in human AAA and adjacent control tissues (*n* = 3), α‐SMA (green), SDC4 and paxillin (red), and DAPI (blue) (scale bars, 250 μm and 100 μm) (magnified photographs). (E) Representative immunofluorescence images of SDC4 and paxillin in control and AngII‐induced AAA mouse samples (*n* = 5); α‐SMA (green), SDC4 and paxillin (red), and DAPI (blue) (scale bars, 300 μm and 100 μm) (magnified photographs)

It has been widely reported that the syndecan family has a close association with cardiovascular disease. We first examined the expression of SDC1–4 in human AAA samples by western blotting (Figure 1A). The clinical data of the patients are listed in Table S1. Compared with those in the control adjacent aorta, the protein levels of Syndcan1 (SDC1) and Syndecan2 (SDC2) were significantly higher in AAA tissues, while the level of SDC4 was significantly reduced. In addition, the protein level of Syndecan3 (SDC3) was barely detectable in AAA and control adjacent aortic tissues (Figure 1A). The role of SDC1 and SDC2 in AAA has been reported,^14^, ^15^ so the present study focused on SDC4. The protein levels of SDC4 and paxillin, two critical molecules in the focal adhesion complex, were distinctly lower in human AAA tissues than in adjacent non‐AAA tissues (Figure 1A,B,D). Moreover, the protein levels of matrix metalloproteinase 2 (MMP2) and MMP9, which promote AAA formation,^16^ were distinctly higher in AAA tissues than in adjacent non‐AAA tissues (Figure 1B). In addition, the degradation of elastic fibres in human AAA tissue was more severe than that in adjacent non‐AAA tissue (Figure S1A). Consistent with those in human AAA samples, the levels of SDC4 and paxillin in mouse AAA tissue were significantly lower than those in normal vascular tissue (Figure 1C,E; Figure S1B). These results suggested that SDC4 downregulation occurred during the development of AAA.

### 3.2 SDC4 downregulation promoted angiotensin II (AngII)‐induced AAA formation in mice

**FIGURE 2:**
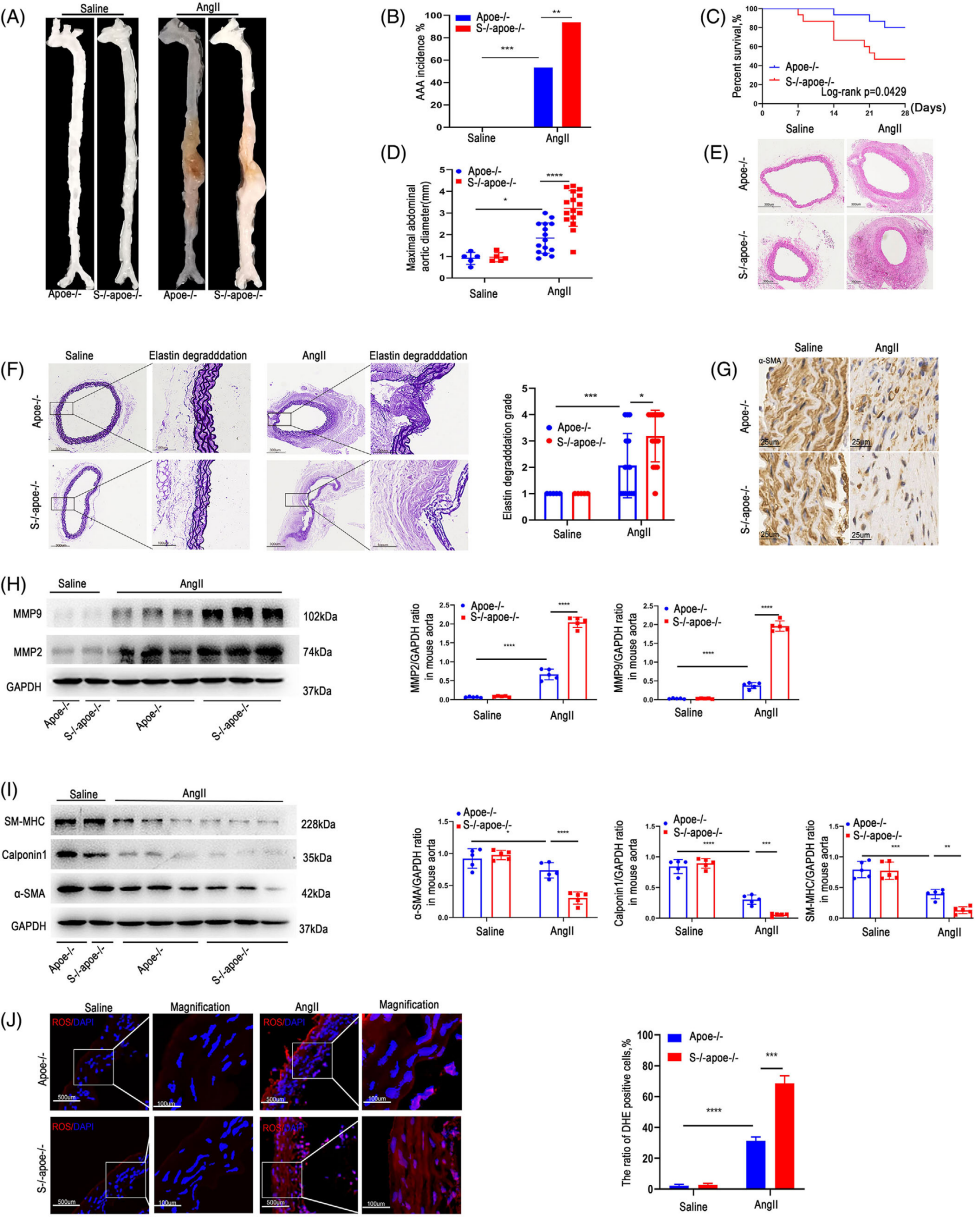
Knockout (KO) of SDC4 promoted AAA formation under Ang II stimulation. All mice were administered AngII or saline for 28 days. (A) Representative images showing the macroscopic features of AngII‐induced AAAs. (B and C) The AAA incidence (B) and survival curve (C) of Ang II‐induced apoe‐/‐ mice (*n* = 15) compared with SDC4‐/‐apoe‐/‐ mice (*n* = 16). Saline infusion did not induce AAA formation (*n* = 10). (D) Statistical analysis of the maximal abdominal aortic diameter in Ang II‐ and saline‐infused mice. (E and F) Representative photographs of H&E staining (E), elastica van Gieson (EVG) staining and elastin degradation scores (F) in abdominal aortas from AngII‐ and saline‐infused mice (*n* = 5) (scale bars, 300 μm and 100 μm) (magnified photographs). (G) Immunohistochemical analysis of α‐SMA‐positive cells in SDC4‐/‐apoe‐/‐ and apoe‐/‐ mice after AngII or saline treatment (scale bars, 25 μm). (H) Representative western blots of MMP9 and MMP2 and densitometric analysis of AngII‐ and saline‐infused mice (*n* = 5). (I) Representative western blots of α‐SMA, Calponin1 and SM‐MHC and densitometric analysis of saline‐ and AngII‐infused mice (*n* = 5). (J) The level of reactive oxygen species (ROS) in the abdominal aortas of AngII‐ and saline‐infused mice was evaluated by dihydroethidium (DHE) staining and quantified by determining the ratio of DHE‐positive cells (*n* = 5) (scale bars, 500 μm and 100 μm) (magnified photographs)

To explore the relationship between SDC4 and AAA formation, we bred SDC4‐/‐apoe‐/‐ double‐knockout (KO) mice by crossing apoe‐/‐ mice with SDC4‐/‐ mice. SDC‐4‐deficient mice have been previously shown to have no obvious defects, exhibiting normal growth, reproduction and tissue morphology.^17^ Hyperlipidaemia was required in the AngII‐induced AAA mouse model. In a previous study, infusion of AngII into apoe+/+ mice failed to generate aneurysms.^18^ Meanwhile, only male mice were enrolled in this study because female mice have a low incidence of Ang II‐induced AAA.^13^ Here, 8‐ to 10‐week‐old male apoe ‐/‐ mice (*n* = 15) and male SDC4‐/‐apoe‐/‐ mice (*n* = 16) were administered the same dose of AngII (1000 ng/kg/min)^19^ for 4 weeks. Another 5 apoe‐/‐ and 5 SDC4‐/‐apoe‐/‐ male mice were infused with the same amount of normal saline for 4 weeks as controls. No AAA was observed in the saline group (Figure 2A,E). In the AngII group, the AAA incidence in SDC4‐/‐apoe ‐/‐ mice was 93.75% (15/16), which was significantly higher than that in apoe ‐/‐ mice (53.3%, 8/15) (Figure 2B). Among the mice infused with AngII, 50% (8/16) of SDC4‐/‐apoe‐/‐ mice and 20% (3/15) of apoe‐/‐ mice died from aortic rupture (Figure 2C). In addition, the maximum AAA diameter (Figure 2D) and the degradation of elastic fibres (Figure 2F) in the vascular wall of AngII‐induced SDC4‐/‐apoe‐/‐ mice were significantly higher than those of apoe‐/‐ mice. Immunohistochemical analysis showed that the number of α‐SMA‐positive cells in the vascular wall of SDC4‐/‐apoe‐/‐ mice stimulated with AngII was significantly lower than that of apoe‐/‐ mice stimulated with AngII (Figure 2G). In the AngII groups, the expression of MMP2 and MMP9 in SDC4‐/‐apoe‐/‐ mice was also significantly higher than that in apoe‐/‐ mice (Figure 2H). The expression levels of the VSMC contractile markers α‐SMA, Calponin1 and SM‐MHC in SDC4‐/‐apoe‐/‐ mice were significantly lower than those in apoe‐/‐ mice (Figure 2I).

In the saline group, there were no significant differences in vascular morphology, maximum diameter or elastic fibre degradation fraction between the two groups. Moreover, the MMP2, MMP9, α‐SMA, Calponin1 and SM‐MHC expression levels did not differ between SDC4‐/‐apoe‐/‐ and apoe‐/‐ mice in the saline group (Figure 2A‐I). Reactive oxygen species (ROS) are key signalling molecules that play important roles in the progression of inflammatory disorders,^20^ so we examined ROS levels in the abdominal aortas of AngII‐ and saline‐infused mice. As shown in Figure 2J, no differences in ROS levels were observed between the saline‐treated Apoe‐/‐ and SDC4‐/‐apoe‐/‐ mice, and ROS accumulation in the vascular wall in SDC4‐/‐apoe‐/‐ mice was more severe than that in apoe‐/‐ mice after AngII stimulation. Taken together, these results suggest that silencing of the SDC4 gene is associated with an increase in AngII‐induced AAA formation.

### 3.3 SDC4 downregulation increased calcium chloride (CaCl_2_)‐induced AAA formation in mice

**FIGURE 3:**
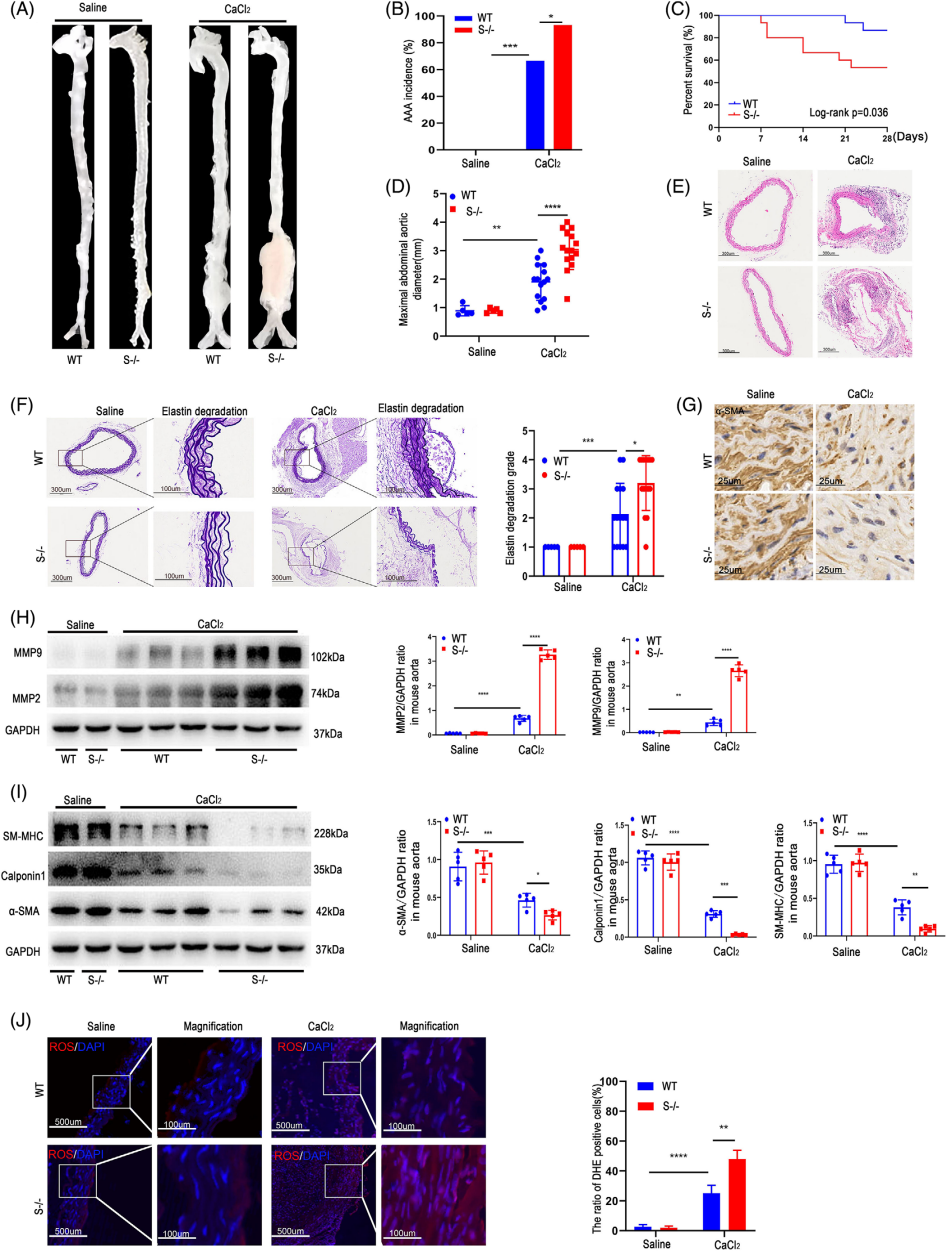
SDC4 KO promoted CaCl_2_‐induced abdominal aortic aneurysm (AAA) formation. (A) Representative images showing the morphology of CaCl_2_‐induced AAAs. (B and C) The AAA incidence (B) and survival curve (C) of wild‐type (WT) mice stimulated with CaCl_2_ (*n* = 15) compared with that of SDC4‐/‐mice (*n* = 15). No AAA developed in saline‐treated mice (*n* = 10). (D) The maximal abdominal aortic diameter in CaCl_2_‐ and saline‐treated mice. (E and F) Representative images of H&E staining (E), elastica van Gieson (EVG) staining and the elastin degradation score (F) of abdominal aortas from CaCl_2_‐ and saline‐treated mice (*n* = 5) (scale bars, 300 μm and 100 μm) (magnified photographs). (G) Immunohistochemical analysis of α‐SMA positive cells in SDC4‐/‐ and WT mice after CaCl_2_ or saline treatment (scale bars, 25 μm). (H) Representative western blots of MMP9 and MMP2 and densitometric analysis of saline‐ and CaCl_2_‐treated mice (*n* = 5). (I) Representative western blots of α‐SMA, Calponin1 and SM‐MHC and densitometric analysis of saline‐ and CaCl_2_‐treated mice (*n* = 5). (J) The level of reactive oxygen species (ROS) in the abdominal aortas of CaCl_2_‐ and saline‐infused mice was evaluated by dihydroethidium (DHE) staining and quantified by determining the ratio of DHE‐positive cells (*n* = 5) (scale bars, 500 μm and 100 μm) (magnified photographs)

To further investigate the role of SDC4 in AAA formation, we used the CaCl_2_‐induced AAA model. Male wild‐type (WT) mice (*n* = 15) and SDC4‐/‐ mice (*n* = 15) were administered CaCl_2_ and sacrificed 4 weeks later. Saline was used as the control. The results showed that there was no occurrence of AAA and no obvious morphological changes in the blood vessels in the saline groups (Figure 3A,B,E). However, in the CaCl_2_ groups, the incidence of AAA in SDC4‐/‐ mice was 93% (14/15), of which nearly 47% (7/15) died from aortic rupture. On the other hand, the incidence of AAA in WT mice was 67% (10/15), and the proportion of mice that died from AAA rupture was approximately 13% (2/15) (Figure 3B,C). Moreover, the maximum diameter and elastic fibre degradation of AAA in WT mice were significantly lower than those in SDC4‐/‐ mice (Figure 3D,F). Immunohistochemical analysis showed that the number of α‐SMA‐positive cells in the vascular wall in CaCl_2_‐induced SDC4‐/‐ mice was significantly lower than that in WT mice (Figure 3G). Consistent with the AngII‐induced AAA model results, the levels of MMP2 and MMP9 were increased in SDC4‐/‐ mice compared with WT mice (Figure 3H). The protein levels of α‐SMA, Calponin1, and SM‐MHC in WT mice were markedly higher than those in SDC4‐/‐ mice (Figure 3I). Additionally, SDC4 KO exacerbated CaCl_2_‐induced ROS accumulation in mice (Figure 3J). These results showed that compared with WT mice, genetic ablation of SDC4 further exacerbated AAA in the CaCl_2_‐induced AAA model.

### 3.4 SDC4 deletion resulted in the transition from a contractile to secretory phenotype in cultured VSMCs

**FIGURE 4:**
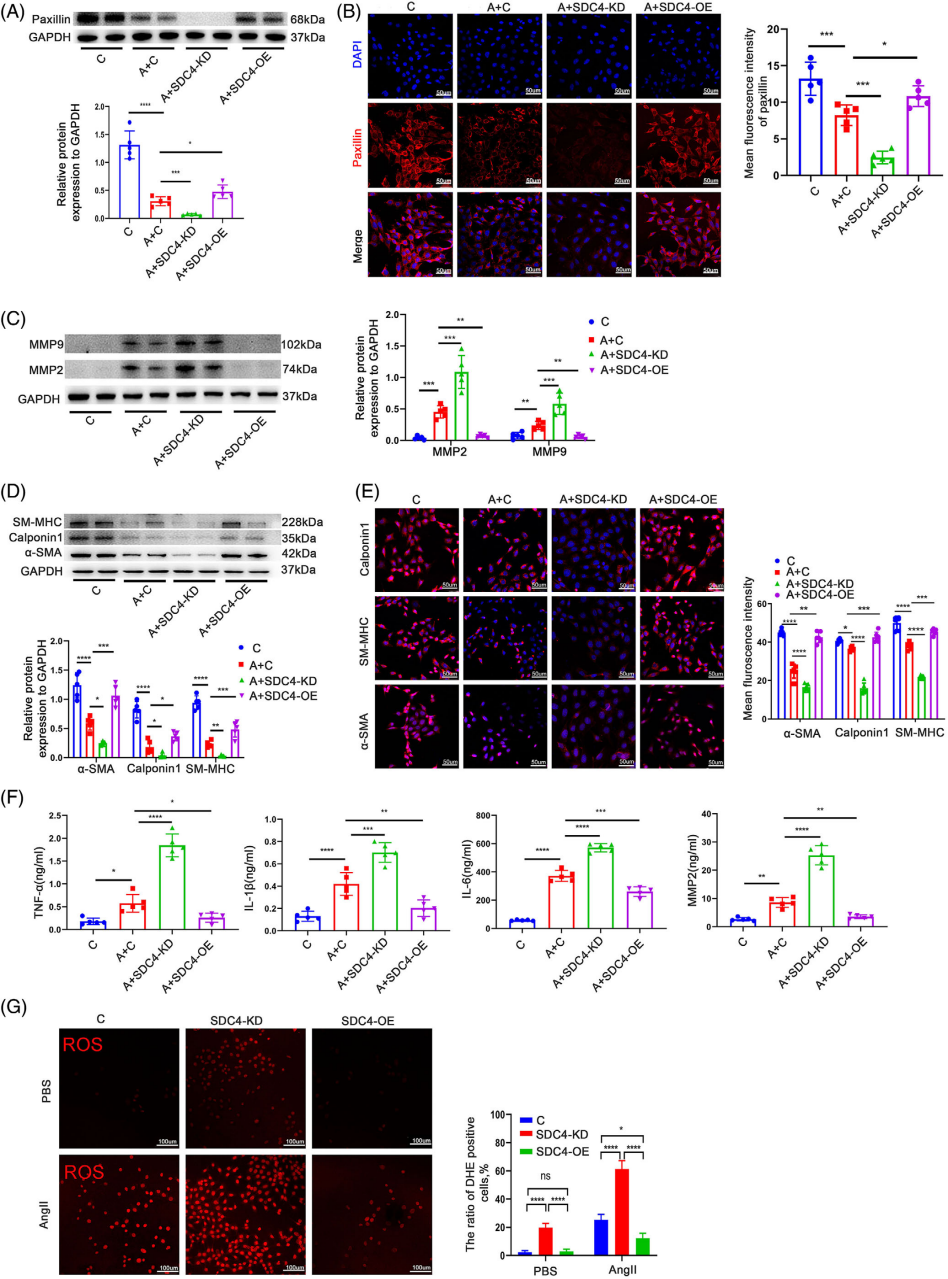
SDC4 altered the phenotype of vascular smooth muscle cells (VSMCs). (A) Representative western blots of paxillin and densitometric analysis of control, AngII+control, AngII+SDC4‐KD and AngII+SDC4‐OE cells (*n* = 5). (B) Immunofluorescence staining and mean fluorescence intensity of paxillin in control, AngII+control, AngII+SDC4‐KD and AngII+SDC4‐OE cells (*n* = 5) (scale bars, 50 μm). (C) Representative western blots of MMP2, MMP9 and densitometric analysis of control, AngII+control, AngII+SDC4‐KD and AngII+SDC4‐OE cells (*n* = 5). (D) Representative western blots of α‐SMA, Calponin1 and SM‐MHC in control, AngII+control, AngII+SDC4‐KD and AngII+SDC4‐OE cells (*n* = 5). (E) Representative immunofluorescence images and mean fluorescence intensity of α‐SMA, Calponin1 and SM‐MHC in control, AngII+control, AngII+SDC4‐KD and AngII+SDC4‐OE cells (*n* = 5) (scale bars, 50 μm). (F) The levels of MMP2, IL‐β, tumour necrosis factor‐α (TNF‐α) and IL‐6 in the supernatants of control, AngII+control, AngII+SDC4‐KD and AngII+SDC4‐OE cells were determined by enzyme linked immunosorbent assay (ELISA) (*n* = 5). (G) The levels of reactive oxygen species (ROS) in control, SDC4‐KD and SDC4‐OE cells with or without AngII stimulation were detected by dihydroethidium (DHE) staining and quantified by determining the ratio of DHE‐positive cells (*n* = 5) (scale bars, 100 μm)

An alteration in the VSMC phenotype is a critical pathological process in AAA formation. In animal models, we observed that contractile markers in VSMCs were significantly decreased in SDC4‐KO mice. Here, we performed in vitro experiments using mouse aortic SMCs to observe the role of SDC4 in VSMC phenotype switching. We constructed SDC4 knockdown (SDC4‐KD) and SDC4 overexpression (SDC4‐OE) lentiviruses to infect VSMCs. qPCR and western blot analysis were used to verify the KD and OE efficiency (Figure S2). As shown in Figure 4A,B, the protein expression of paxillin was significantly lower in AngII+SDC4‐KD cells than in AngII+control cells, indicating that SDC4 KD reduced the level of paxillin. However, the level of paxillin expression in AngII+SDC4‐OE cells was higher than that in AngII+control cells, which showed that SDC4 OE in VSMCs protects against the effects of AngII. Under physiological conditions (without AngII stimulation), SDC4 KD in VSMCs also reduced the level of paxillin, while no difference was observed between SDC4‐OE cells and control cells (Figure S3A,B). Then, we measured the expression of the secretory phenotypic markers MMP2 and MMP9 and the contractile markers α‐SMA, Calponin1 and SM‐MHC by western blotting and immunofluorescence. The levels of MMP2 and MMP9 were higher, and the levels of α‐SMA, Calponin1 and SM‐MHC were lower in AngII +SDC4‐KD cells than in AngII+control cells. SDC4 OE rescued these molecular alterations in VSMCs after AngII stimulation compared with AngII+control cells (Figure 4C‐E). Under physiological conditions (without AngII stimulation), SDC4 KD also induced the expression of MMP2 and MMP9 (Figure S3C,D) and reduced the levels of α‐SMA, Calponin1 and SM‐MHC (Figure S3E‐J). No difference was observed in the levels of MMP2, MMP9, α‐SMA, Calponin1 or SM‐MHC between SDC4‐OE cells and control VSMCs (Figure S3A‐J). These results indicated that SDC4 OE alleviated AngII‐induced phenotypic switching in VSMCs and that the deletion of SDC4 in VSMCs led to a secretory phenotype.

A chronic inflammatory response was observed in AAA tissues.^5^ Secretory VSMCs have been reported to be a source of several inflammatory cytokines.^21^ Here, we collected the supernatants of control, AngII+control, AngII+SDC4‐KD and AngII+SDC4‐OE cells and measured the levels of the inflammatory cytokines interleukin‐1β (IL‐1β), interleukin‐6 (IL6) and tumour necrosis factor‐α (TNF‐α), as well as the secreted protein MMP2, in these groups. The results demonstrated that AngII increased the levels of IL‐1β, IL6, TNF‐α and MMP2 compared with those in control cells. The levels of these molecules in AngII+SDC4‐KD cells were significantly higher than those in AngII+control cells, indicating that SDC4‐KD in VSMCs exacerbated the AngII‐induced expression of these molecules. SDC4 OE in VSMCs significantly attenuated the expression of these inflammatory cytokines compared with that in AngII+control cells (Figure 4F). Under physiological conditions (without AngII stimulation), SDC4 KD also increased the expression of these inflammatory cytokines (Figure S3K). In addition, under physiological conditions (PBS treatment), SDC4 KD increased cellular ROS levels, and under pathological conditions (AngII stimulation), SDC4 OE attenuated ROS accumulation in VSMCs (Figure 4G). These data indicated that SDC4 OE in VSMCs reduced the secretion of inflammatory factors and thus played a protective role in preventing AAA formation under pathological conditions.

### 3.5 SDC4 regulated the phenotypic switching of VSMCs through the RhoA‐F/G‐Actin‐myocardin‐related transcription factor‐A pathway

**FIGURE 5:**
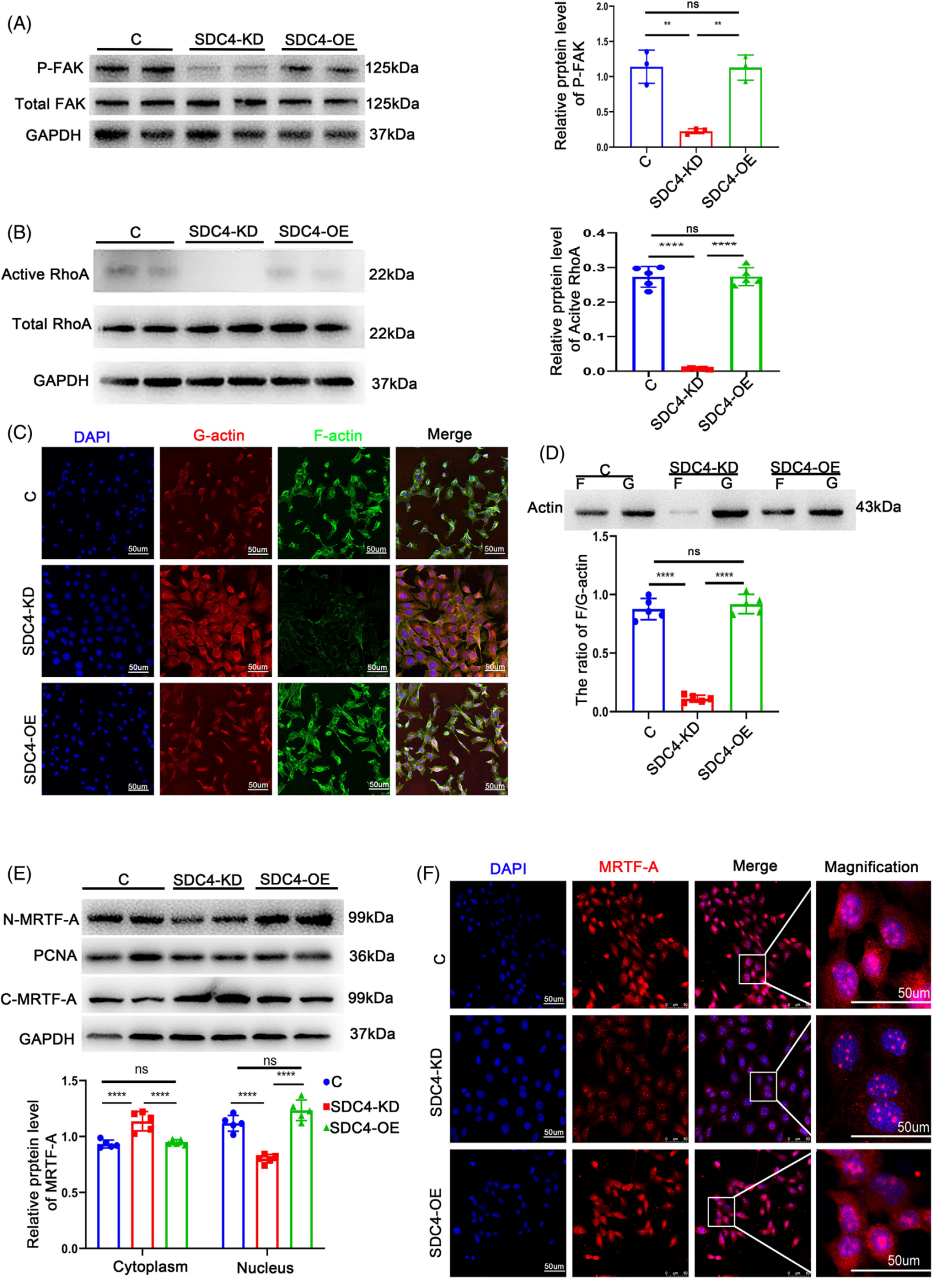
SDC4 was crucial in maintaining the contractile phenotype of vascular smooth muscle cells (VSMCs) via the RhoA‐MRTF‐A axis. (A) Representative western blots of P‐FAK and densitometric analysis of control, SDC4‐KD and SDC4‐OE cells (*n* = 5). (B) Representative western blots of active RhoA and densitometric analysis of control, SDC4‐KD and SDC4‐OE cells (*n* = 5). (C) Representative phalloidin staining of G‐actin and F‐actin in control, SDC4‐KD and SDC4‐OE cells (*n* = 5) (scale bars, 50 μm). (D) Relative protein expression of G‐actin and F‐actin was determined by western blot analysis with a commercial kit (cytoskeleton, Cat #BK037) in control, SDC4‐KD and SDC4‐OE cells (*n* = 5). (E) Representative western blots of MRTF‐A and densitometric analysis of proteins in the cytoplasm and nucleus in control, SDC4‐KD and SDC4‐OE cells (*n* = 5). (F) Representative immunofluorescence images of nuclear MRTF‐A in control, SDC4‐KD and SDC4‐OE cells were analysed by confocal microscopy (*n* = 5, scale bars, 50 μm)

SDC4 has previously been demonstrated to regulate focal adhesion kinase (FAK) phosphorylation, which is known to correlate with the level of autophosphorylation at the Y397 locus^22^; thus, we measured the level of Tyr397‐FAK phosphorylation. As shown in Figure 5A, the level of Tyr397‐FAK phosphorylation was significantly lower in SDC4‐KD cells than in control and SDC4‐OE cells. To further investigate the molecular mechanism of SDC4 in the VSMC phenotype, we examined RhoA activity in VSMCs with or without SDC4 expression. It has been reported that SDC4 phosphorylates RhoGDI via downstream PKCα to promote RhoA activation.^23^ RhoA plays a vital role in F‐actin synthesis and actin dynamic homeostasis and is critical in maintaining the contractile VSMC phenotype through the myocardin‐related transcription factor‐A (MRTF‐A) signalling pathway.^24^ We observed that RhoA activity was decreased after SDC4 deletion (Figure 5B), and the ratio of F/G‐actin was significantly downregulated by SDC4 deletion (Figure 5C,D). However, we found that nuclear expression of MRTF‐A was reduced considerably in SDC4‐KO cells compared with WT and SDC4‐OE cells (Figure 5E,F). These results indicated that the loss of SDC4 altered the RhoA‐F/G‐Actin‐MRTF‐A pathway.

**FIGURE 6:**
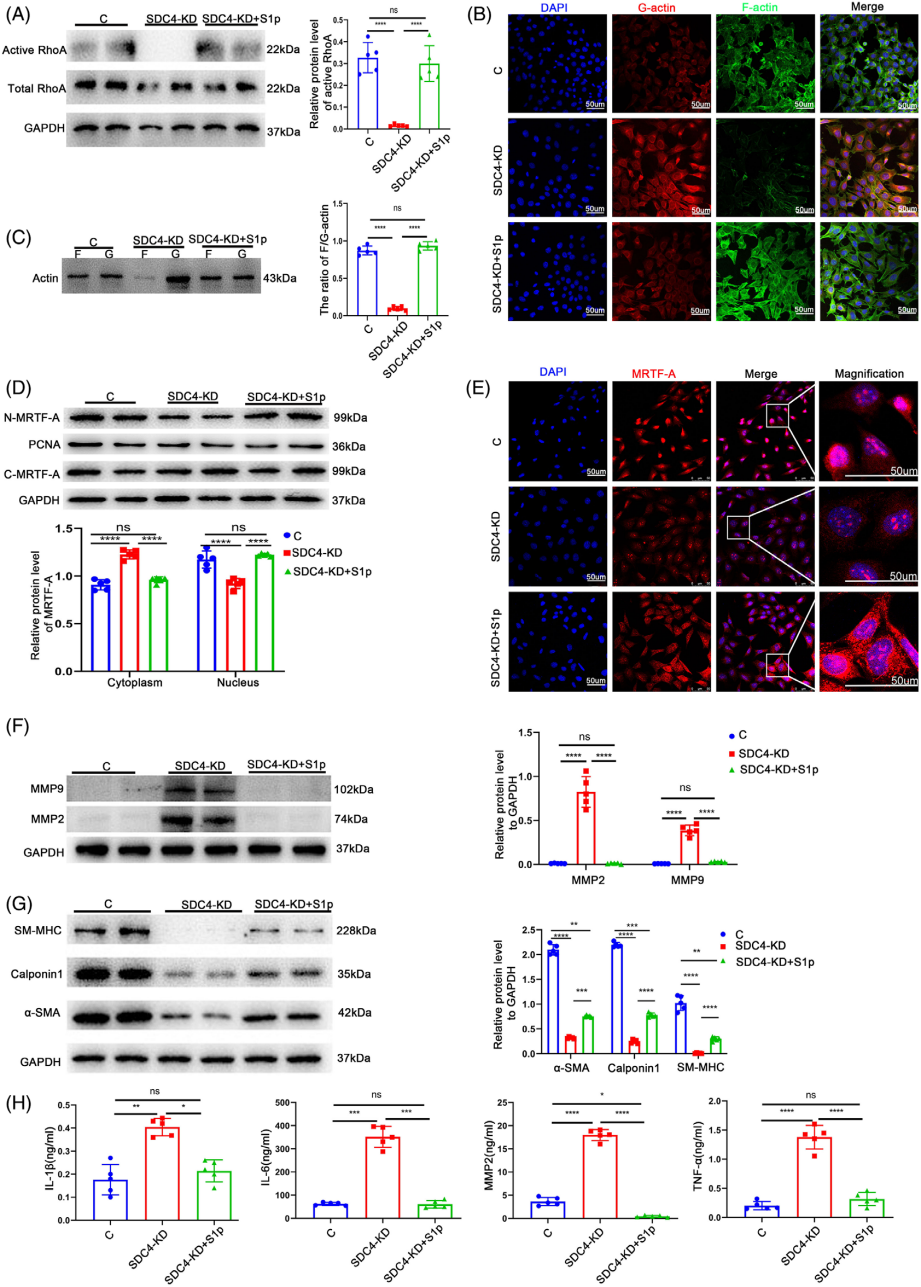
S1p maintained the contractile phenotype and inhibited inflammatory cytokine expression in SDC4‐/‐ cells. (A) Representative western blots of active RhoA and densitometric analysis of control, SDC4‐KD and SDC4‐KD+S1p cells (*n* = 5). (B) Representative phalloidin staining of F‐actin and G‐actin in control, SDC4‐KD, and SDC4‐KD+S1p cells (*n* = 5). (C) Representative western blots showing the ratio of F‐actin/G‐actin and densitometric analysis of control, SDC4‐KD and SDC4‐KD+S1p cells (*n* = 5). (D) Representative western blots of MRTF‐A and densitometric analysis of proteins in the cytoplasm and nucleus in control, SDC4‐KD and SDC4‐KD+S1p cells (*n* = 5). (E) Representative immunofluorescence images showing the expression of MRTF‐A in control, SDC4‐KD and SDC4‐KD+S1p cell nuclei (*n* = 5, scale bars, 50 μm). (F) Representative western blots of MMP9 and MMP2 and densitometric analysis of control, SDC4‐KD and SDC4‐KD+S1p cells (*n* = 5). (G) Representative western blots of α‐SMA, Calponin1 and SM‐MHC and densitometric analysis of control, SDC4‐KD and SDC4‐KD +S1p cells (*n* = 5). (H) The levels of MMP2, IL‐β, tumour necrosis factor‐α (TNF‐α) and IL‐6 in the supernatants of control, SDC4‐KD and SDC4‐ KD+S1p cells were determined by ELISA (*n* = 5)

To further determine whether the loss of SDC4 changed the phenotype of VSMCs via the RhoA‐F/G‐Actin‐MRTF‐A pathway, the GPCR agonist sphingosine‐1‐phosphate (S1p),^25^ which can activate RhoA, was added to SDC4‐KD cells, and phenotypic alterations were observed. After the addition of S1P (1 μg/ml, 10 min) to SDC4‐KD cells, the proportion of activated RhoA increased significantly (Figure 6A), accompanied by significant recovery of F/G‐actin (Figure 6B,C), and the ratio of MRTF‐A in the nucleus also increased notably (Figure 6D,E). Interestingly, compared with that in SDC4‐KO cells, the expression of MMP2 and MMP9 in SDC4‐KO +S1p cells was significantly decreased after S1p treatment (Figure 6F), and the contractile VSMC markers α‐SMA, Calponin1 and SM‐MHC were also significantly increased (Figure 6G and Figure S4). In addition, the levels of the inflammatory cytokines IL‐6, IL‐1β and TNF‐α and the secreted protein MMP2 were also significantly reduced after S1p treatment compared with those in SDC4‐KO cells (Figure 6H).

To further prove that the phenotype of SMCs was affected by SDC4 through the RhoA pathway, we added a RhoA inhibitor (2 μg/ml, 4 h, CT04, a cell‐permeable C3 transferase, cytoskeleton) to SDC4‐OE cells and then observed the phenotypic change in SMCs. The expression levels of α‐SMA, Calponin1 and SM‐MHC were significantly decreased, while those of MMP2 and MMP9 were notably increased in SDC4‐OE +CT04 cells compared with control and SDC4‐OE cells (Figure S5A‐D). Moreover, the levels of IL‐6, IL‐1β, TNF‐α and MMP2 were also significantly increased after CT04 treatment compared with those in control and SDC4‐OE cells (Figure S5E). These results indicated that the SDC4 mediates the phenotype of SMCs via the RhoA‐depended pathway.

### 3.6 CYM‐5478, an S1p analogue, alleviated the development of AAA in mice

**FIGURE 7:**
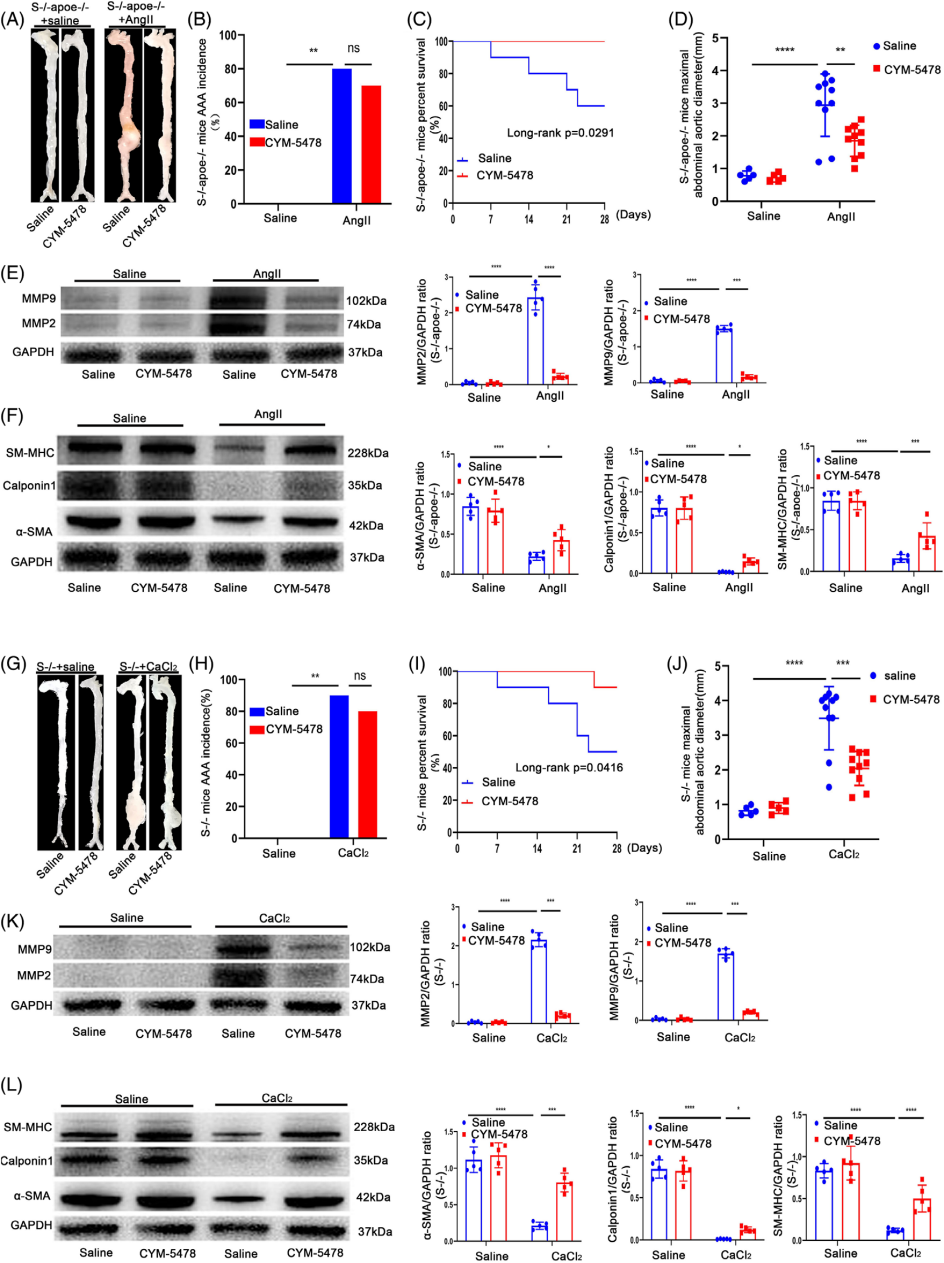
CYM‐5478 delayed the progression of AAA in SDC4‐/‐ mice. All mice were treated with CYM‐5478 or saline for 28 days. (A) Representative images showing the macroscopic features of AngII‐induced AAAs. (B and C) abdominal aortic aneurysm (AAA) incidence (B) and survival curve (C) of Ang II‐induced SDC4‐/‐ apoe‐/‐+saline mice (*n* = 10) compared with SDC4‐/‐ apoe‐/‐ + CYM‐5478 mice (*n* = 10). (D) Statistical analysis of the maximal abdominal aortic diameters in Ang II‐ and saline‐infused mice. (E) Representative western blots of MMP9 and MMP2 and densitometric analysis of AngII‐ and saline‐infused mice with or without CYM‐5478 treatment. (F) Representative western blots of α‐SMA, Calponin1 and SM‐MHC and densitometric analysis of AngII‐ and saline‐infused mice with or without CYM‐5478 treatment (*n* = 5). (G‐J) Morphology (G), AAA incidence (H), survival curve (I) and maximal abdominal aortic diameter (J) of CaCl_2_‐induced SDC4‐/‐+saline mice (*n* = 10) compared with SDC4‐/‐ + CYM‐5478 mice (*n* = 10). (K) Representative western blots of MMP9 and MMP2 and densitometric analysis of CaCl_2_‐ and saline‐infused mice with or without CYM‐5478 treatment. (L) Representative western blots of α‐SMA, Calponin1 and SM‐MHC and densitometric analysis of CaCl_2_‐ and saline‐infused mice with or without CYM‐5478 treatment (*n* = 5)

**FIGURE 8:**
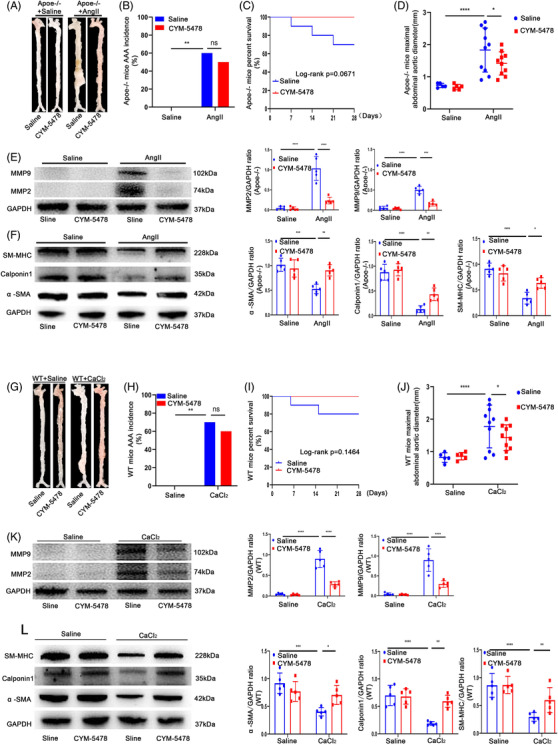
CYM‐5478 delayed the progression of abdominal aortic aneurysm (AAA) in wild‐type (WT) mice. (A) Representative images showing the macroscopic features of AngII‐induced AAAs. (B and C) AAA incidence (B) and survival curve (C) of Ang II‐induced Apoe‐/‐+saline mice (*n* = 10) compared with Apoe‐/‐ + CYM‐5478 mice (*n* = 10). (D) Statistical analysis of the maximal abdominal aortic diameter in Ang II‐ and saline‐infused mice. (E) Representative western blots of MMP9 and MMP2 and densitometric analysis of AngII‐ and saline‐infused mice with or without CYM‐5478 treatment. (F) Representative western blots of α‐SMA, Calponin1 and SM‐MHC and densitometric analysis of AngII‐ and saline‐infused mice with or without CYM‐5478 treatment (*n* = 5). (G‐J) Morphology (G), AAA incidence (H), survival curve (I) and maximal abdominal aortic diameter (J) of CaCl_2_‐induced WT+saline mice (*n* = 10) compared with WT + CYM‐5478 mice (*n* = 10). (K) Representative western blots of MMP9 and MMP2 and densitometric analysis of CaCl_2_‐ and saline‐infused mice with or without CYM‐5478 treatment. (L) Representative western blots of α‐SMA, Calponin1 and SM‐MHC and densitometric analysis of CaCl_2_‐ and saline‐infused mice with or without CYM‐5478 treatment (*n* = 5)

To verify whether SDC4 also acts through the RhoA pathway in vivo, SDC4‐/‐ apoe‐/‐male mice were randomly divided into two groups. One group was supplemented daily with CYM‐5478 (MolPort‐004‐121‐217, MolPort, Latvia, 1 mg/kg),^26^ a specific agonist of the S1P2 receptor, which is the best‐characterized effector pathway of S1P2 that activates the small GTPase RhoA through Gα12 and Gα13,^27^ and the other group was supplemented with an equal amount of normal saline. All of the mice were infused with AngII, and the occurrence of AAA was observed 28 days later. The results demonstrated that there was no statistically significant difference in the incidence of AAA between the two groups (Figure 7B), but the maximum AAA diameter and the number of mice that died from aortic rupture were significantly lower in the CYM‐5478 group than in the saline group (Figure 7A,C,D). The expression levels of MMP2 and MMP9 were significantly decreased, and the contractile VSMC markers α‐SMA, Calponin1 and SM‐MHC were also significantly increased after CYM‐5478 treatment compared with saline treatment in the AngII group (Figure 7E,F). Moreover, CYM‐5478 also reduced the maximum AAA diameter and improved the survival rate of CaCl_2_‐induced AAA model mice, reduced the levels of MMP2 and MMP9 (Figure 7G‐K) and increased the levels of α‐SMA, Calponin1 and SM‐MHC compared with saline treatment in the CaCl_2_ group (Figure 7L). These data indicated that CYM‐5478 alleviated the development of AAA in SDC4‐KO mice. Moreover, we selected 20 male apoe‐/‐ mice, which were randomly divided into two groups, and induced AAA with AngII. One group was supplemented with CYM‐5478, and the other group was supplemented with an equal amount of normal saline. Twenty‐eight days later, there was no significant difference in the incidence of AAA or the percent survival of mice between the two groups. However, the maximum AAA diameter in the CYM‐5478 group was significantly lower than that in the saline group (Figure 8A‐D). CYM‐5478 also reduced the levels of MMP2 and MMP9 and increased the levels of α‐SMA, Calponin1 and SM‐MHC compared with saline treatment in the AngII group (Figure 8E,F). Interestingly, we obtained the same results in CaCl_2_‐induced WT mice (Figure 8G‐L). These data indicated that CYM‐5478 alleviated the development of AAA in WT mice. Next, we examined whether CYM‐5478 also exerted a protective effect after AAA formation in mice. Unfortunately, there was no significant difference in the survival rate, AAA incidence or maximal abdominal aortic diameter after 2 weeks of CYM5478 treatment compared with saline treatment after AAA formation (Figure S6). These results demonstrated that CYM‐5478 could be used as a potential agent to alleviate the development of AAA in mice.

## 4 DISCUSSION

In the present study, we demonstrated that SDC4 could prevent phenotypic changes in VSMCs, thereby delaying the onset of AAA. There are several main findings in this study. First, the levels of SDC4 were significantly reduced in human AAA samples. Second, SDC4 KO in vivo enhanced AngII‐induced and CaCl_2_‐induced AAA formation in mice. Third, SDC4 downregulation led to a change in VSMCs from the contractile to the secretory phenotype. Finally, the RhoA‐F/G‐actin‐MRTF‐A pathway was involved in this process, and administration of S1p slowed the development of AAA.

Here, we observed that the SDC4 protein level was decreased in human and murine AAA samples. In the aortic walls of normal mice, we observed that SDC4 but not SDC1–3 localized to VSMCs in the media. On the other hand, previous studies reported that SDC1 was predominantly located in macrophages and that SDC2 was abundantly expressed in arterial thrombi. SDC3 was primarily localized to nerve cells, which were hardly observed in aortic tissue.^15^, ^28^ Hence, we focused on the role of SDC4 in the development of AAA.

As a non‐specific cell membrane receptor, SDC4 is not static.^29^ During chronic inflammation, IL‐1β, TNF‐α and lipopolysaccharide induce shedding of the SDC4 ectodomain, affecting SDC4 and its downstream signalling pathways.^30^, ^31^ In turn, a reduction in SDC4 further exacerbates vascular inflammation by altering the VSMC phenotype. Furthermore, a key mechanism associated with AAA pathogenesis is the production of ROS and oxidative stress.^32^ Houston et al. reported that after ROS exposure, a continuous reduction in both SDC4 protein and mRNA levels was subsequently observed.^33^

AAA is characterized as a permanent localized dilation of the abdominal aortic wall that exceeds 50% of the normal vessel diameter.^2^ The transformation of VSMCs from the contractile phenotype to the secretory phenotype is the key event in the pathological process of AAA, which is directly affected by the development and progression of chronic inflammation and vascular matrix remodelling. VSMCs with the secretory phenotype lose their contractile functions, and many MMP‐related proteins and abnormal collagen proteins accumulate, resulting in a decrease in local vascular elasticity and a great increase in the risk of aneurysm formation.^34^ VSMC phenotypic changes are of great value in AAA and have gradually received increased attention in recent years, but the related research progress has been slow, and the mechanism is still unclear. This study found that SDC4 downregulation led to a decrease in RhoA activity, resulting in reductions in the proportion of downstream F/G‐actin and MRTF‐A in the nucleus. MRTF‐A is a crucial transcription factor that is present in the cytoplasm by binding to G‐actin.^35^ When G‐actin is polymerized into F‐actin, MRTF‐A is uncoupled from G‐actin and enters the nucleus, forming a ternary correspondence with serum response factor and the CArG frame to activate the transcription of VSMC‐specific promoters.^36^ It has been reported that abnormalities in the MRTF‐A pathway lead to the downregulation of α‐SMA, Calponin1 and SM‐MHC expression and affect the VSMC contractile phenotype.^36^, ^37^ Accumulating evidence has shown that SDC4 plays a crucial role in maintaining the VSMC phenotype via the RhoA‐MRTF‐A signalling pathway.

Chronic inflammation is the main entry point in the current study of AAA and is considered to be the key pathogenic cause of AAA formation.^38^ Here, we examined the levels of SDC4 in human AAA samples and mouse AAA models and showed that these levels were significantly reduced under chronic inflammation. Moreover, SDC4 KD exacerbated ROS accumulation in the vasculature of AngII‐ and CaCl_2_‐induced AAA model mice. In vitro, SDC4 deficiency led to a switch from contractile to secretory VSMCs, which secrete a large number of inflammatory factors, leading to the persistence of the inflammatory response in the vascular media. On the other hand, SDC4 OE in VSMCs under pathological conditions significantly inhibited the secretion of inflammatory factors by VSMCs and reduced vascular inflammation. To our knowledge, this is the first report showing that a decrease in SDC4 leads to phenotypic changes in VSMCs and increases the secretion of vascular inflammatory factors, thus promoting AAA formation. These findings have indicated a specific link between SDC4 and AAA, providing a new target for clinical drug research.

There are some limitations to our research. As a membrane receptor, SDC4 is expressed on many cells in the aortic. The present study focused on the role of SDC4 in VSMCs. Whether the loss of SDC4 affects other cells during AAA formation needs to be further explored. In this study, SDC4 and Apoe double‐KO mice were required to establish an AngII‐induced AAA model. Although cre‐loxp‐dependent VSMC‐specific SDC4 KO would provide more definitive evidence, it is too difficult to obtain VSMC‐specific SDC4 and Apoe double‐KO mice. Hence, we used global SDC4 and Apoe KO mice in our animal study, which caused some limitations for our research.

## 5 CONCLUSION

The loss of SDC4, a focal adhesion component of VSMCs, is associated with the development of AAA and serves as a notable target for AAA drug research and development.

## CONFLICT OF INTEREST

The authors have declared no conflict of interest.

## Supporting information



Supporting Information

Supporting Information

Supporting Information

Supporting Information

Supporting Information

Supporting Information

Supporting Information
