# Supplementary material for: A reduction in the vascular smooth muscle cell focal adhesion component syndecan‐4 is associated with abdominal aortic aneurysm formation
Source: Clin Transl Med. 2021 Dec 22;11(12):e605. doi: 10.1002/ctm2.605 (PMC8693440; doi:10.1002/ctm2.605)
Supplement: Supplementary file 1 — Supporting Information [file CTM2-11-e605-s001.docx]

**A Reduction in the Vascular Smooth Muscle Cell Focal Adhesion Component Syndecan-4 is Associated with Abdominal Aortic Aneurysm Formation**

Running title：Syndecan-4 links the formation of AAA

Jiaxin Hu^a #^, Yuyu Li^a #^, Zhonghai Wei^a #^, Haiting Chen^a^, Xuan Sun^a^, Qing Zhou^b^, Qi Zhang ^a^, Yong Yin^a^, Meng Guo^a^, Jianzhou Chen^a^,Guangyao Zhai^c^, Biao Xu^a *^, Jun Xie^a *^

^a^ MOE Key Laboratory of Model Animal for Disease Study, Department of Cardiology, Nanjing Drum Tower Hospital, Model Animal Research Center, School of Medicine, Nanjing University, Nanjing, 210061, China

^b^ Department of Cardiac Surgery, Drum Tower Hospital, Medical School of Nanjing University, Nanjing, China

^c^ Department of Cardiology, Beijing Anzhen Hospital, Capital Medical University, Beijing, China

*Correspondence to Jun Xie and Biao Xu.

Jun Xie, MOE Key Laboratory of Model Animal for Disease Study, Department of Cardiology, Nanjing Drum Tower Hospital, Model Animal Research Center, School of Medicine, Nanjing University, Zhongshan Road, Nanjing, 210008, China. Phone and Fax: 86-25-68182812, E-mail: [xiejun@nju.edu.cn](mailto:xiejun@nju.edu.cn); ORCID no: 0000-0002-9385-3242.

Biao Xu: MOE Key Laboratory of Model Animal for Disease Study, Department of Cardiology, Nanjing Drum Tower Hospital, Model Animal Research Center, School of Medicine, Nanjing University, Zhongshan Road, Nanjing, 210008, China. Phone and Fax: 86-25-68182812, E-mail: xubiao62@nju.edu.cn;

# These authors equally contributed to this article.

**SUPPLEMENTARY FIGURE LEGEND**

**Figure S1 (A)** Elastica van Gieson (EVG) staining in abdominal aortas from human AAA and adjacent control tissues (scale bars, 2mm, and 100 µm) (magnified photographs). (B) Representative immunofluorescence image of SDC4 and paxillin in control and CaCl_2_-induced AAA mouse samples (n=5). α-SMA (green), SDC4 and Paxillin (red), DAPI (blue). (scale bars, 300 µm, and 100 µm) (magnified photographs).

**Figure S2** Identification of SDC4 knockdown and overexpression in mouse aortic vascular smooth muscle cells. (A) Relative mRNA level of SDC4 was detected by RT-PCR(n=3). (B) Relative western blots of SDC4 and densitometry analysis in control and SDC4-KO cells(n=3). (C-D) RT-PCR and Western blots detected the overexpression efficiency of SDC4 in SDC4-OE compared with control cells(n=3). *P < 0.05, **P < 0.01, ***P < 0.001, ****P < 0.0001, ns= not significant.

**Figure S3** SDC4 altered the phenotype of vascular smooth muscle cells. (A) Representative western blots of paxillin and densitometry analysis in control, SDC4-KD and SDC4-OE cells (n=5). (B) Representative immunofluorescence images and mean fluorescence intensity analysis of paxillin in control, SDC4-KD and SDC4-OE cells (n=5). scale bars, 50 µm. (C) Relative MMP2/MMP9 mRNA expression level was detected by real-time PCR in control, SDC4-KO, and SDC4-OE cells(n=3). (D) Representative western blots of MMP2, MMP9, and densitometry analysis in control, SDC4-KO, and SDC4-OE cells (n=5). (E) Relative α-SMA, Calponin1, and SM-MHC mRNA expression level was detected by real-time PCR in control, SDC4-KO, and SDC4-OE cells (n=3). (F) Representative western blots of α-SMA, Calponin1, and SM-MHC and densitometry analysis in control, SDC4-KO, and SDC4-OE cells(n=5). (G-J) Representative immunofluorescence staining and mean fluorescence intensity analysis of α-SMA, Calponin1, and SM-MHC in control, SDC4-KO, and SDC4-OE cells (n=6), scale bars, 50 µm. (K) The levels of MMP2, IL-β，TNF-α and IL-6 in the supernatant of control, SDC4-KO and SDC4-OE cells were detected by ELISA (n=5).*P < 0.05, **P < 0.01, ***P < 0.001, ****P < 0.0001, ns= not significant.

**Figure S4** Immunofluorescence staining detected the expression of α-SMA, Calponin1, and SM-MHC in control, SDC4-KO, and SDC4-KO+S1P cells. (A-D), Representative immunofluorescence staining and mean fluorescence intensity analysis of α-SMA (A), Calponin1 (B), SM-MHC (C) in control, SDC4-KO, and SDC4-KO+S1P cells (n=5), scale bars, 50 µm. *P < 0.05, **P < 0.01, ***P < 0.001, ****P < 0.0001, ns= not significant.

FigureS5

CT04 changed the contractile phenotype and promoted inflammatory cytokine expression in SDC4-OE SMCs cells. (A-B) Representative western blots of MMP9 and MMP2 and densitometric analysis of control, SDC4-OE, and SDC4-OE+CT04 cells (n=5). (C-D) Representative western blots of α-SMA, Calponin1, and SM-MHC and densitometric analysis of control, SDC4-OE, and SDC4-OE +CT04 cells (n=5). (E) The levels of MMP2, IL-β, TNF-α and IL-6 in the supernatants of control, SDC4-OE, and SDC4- OE+CT04 cells were determined by ELISA (n=5).

Figure S6

The role of CYM-5478 in the mice after AAA formation. All mice were treated with AngII and calcium chloride to induce AAA for 28 days, followed by continuous treatment with CYM-5478 for 14 days, and mice were [sacrifice](javascript:;)d after 42 days to observe the AAA relevant changes. (A), Representative images showing the macroscopic features of AngII – induced abdominal aortic aneurysms. (B-C) The AAA incidence (B) and survival curve (C) of Ang II-induced AAA in SDC4-/- apoe-/-+saline mice (n=10) compared with that in SDC4-/- apoe-/- + CYM-5478 mice (n=10). (D) Statistical analysis of the maximal abdominal aortic diameter in Ang II - and saline-infused mice. (E-H), The morphology (E), AAA incidence (F), survival curve (G), and the maximal abdominal aortic diameter (H) of CaCl2-induced AAA in SDC4-/-+Saline mice (n=10) compared with that in SDC4-/- + CYM-5478 mice (n=10). (I-L) The morphology (I), AAA incidence (J), survival curve (K), and the maximal abdominal aortic diameter(L) of AngII-induced AAA in apoe-/- +Saline mice (n=10) compared with that in apoe-/- + CYM-5478 mice (n=10). (M-P) The morphology (M), AAA incidence (N), survival curve (O), and the maximal abdominal aortic diameter (P) of CaCl2-induced AAA in WT+Saline mice (n=10) compared with that in WT+ CYM-5478 mice (n=10).

SUPPLEMENTAL TABLES

Supplemental Table 1 The information of patients

| Number | Age | Gender | AAA size  （cm） |
| --- | --- | --- | --- |
| 1C/A | 54 | Male | 5.5 |
| 2C/A | 67 | Male | 5.3 |
| 3C/A | 45 | Female | 5.0 |

All the samples were obtained from AAA patients, who received an open surgical repair process. AAA size was determined by ultrasound scanning. The control samples were obtained from the adjacent normal region of the aorta from the same patients. All samples are immediately stored in a -80°C refrigerator. C: adjacent control aorta. A: abdominal aortic aneurysm.

Supplemental Table 2 List of primers used for quantitative real‐time PCR


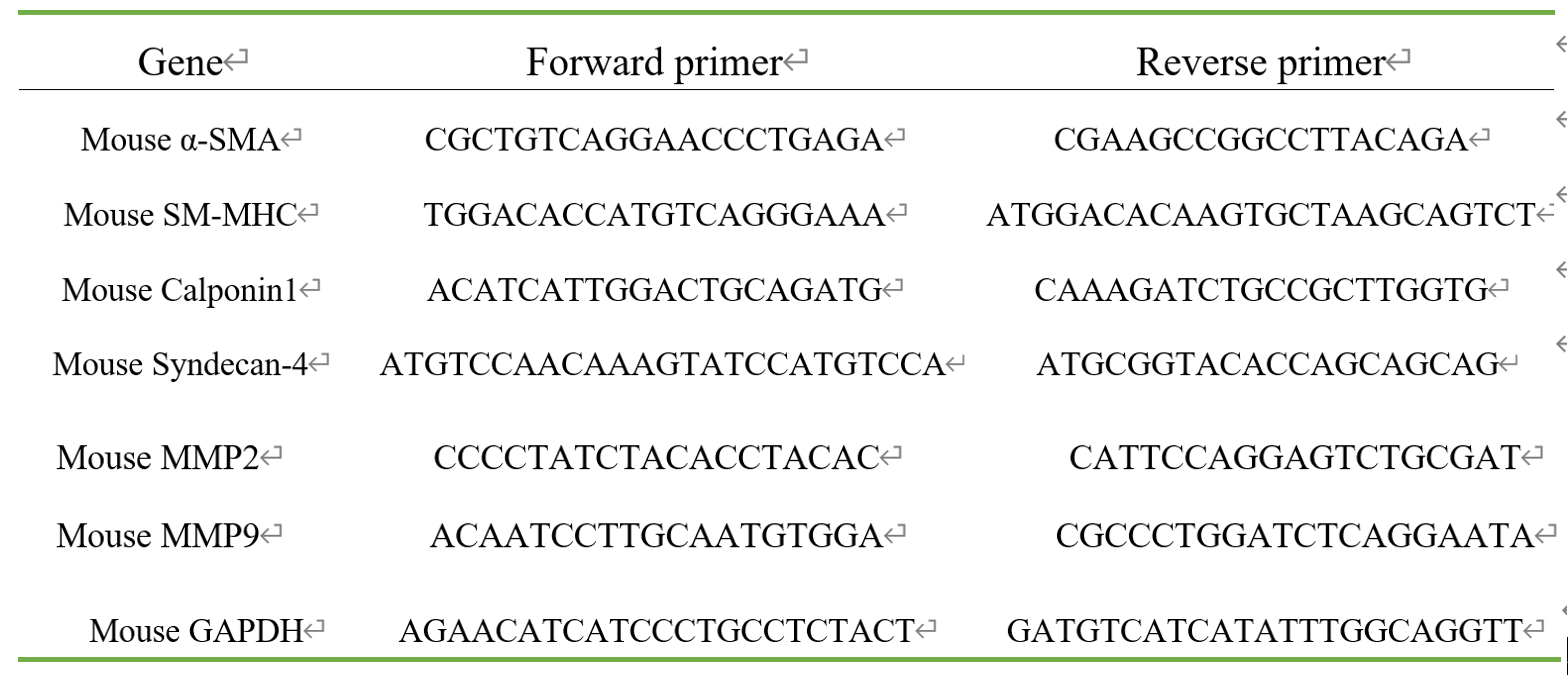


Supplemental Table 3 Primary antibodies used for western blotting

| Antibody | Source | Catalog number | Host species |
| --- | --- | --- | --- |
| Paxillin | Abcam | Ab32084 | rabbit |
| Syndecan-4  Syndecan-1  Syndecan-2  Syndecan-3 | Abcam  Abcam  Santa  Santa | Ab24511  Ab128936  Sc-365624  Sc-398194 | rabbit  rabbit  mouse  mouse |
| Calponin1 | Abcam | Ab46794 | rabbit |
| SM-MHC | Abcam | Ab53219 | rabbit |
| α-SMA | Abcam | Ab5694 | rabbit |
| MMP9 | Abcam | Ab38898 | rabbit |
| MMP2  MRTF-A  PCNA  FAK  Phospho-FAK(Tyr397)  GAPDH | Abcam  Abcam  CST  Abcam  CST  ProMab | Ab97779  Ab115319  #13110  Ab40794  #3283  20035 | rabbit  Rabbit  Rabbit  Rabbit  Rabbit  mouse |


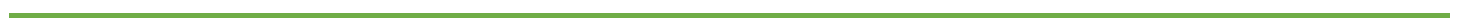


**SUPPLEMENTAL METHODS**

**Animal** **experiment method**

The 8-10-week-old ApoE-/- transgenic mice (purchased from The Model Animal Research Institute of Nanjing University) and SDC4 transgenic mice (SDC4-/-) (owned by our laboratory) were cross-bred to obtain SDC4-/-ApoE-/- transgenic mice.

1. AngII-induced model Hyperlipidemia was required because infusions of Ang II into apoe+/+ mice failed to generate aneurysms. Infusion of AngII into Apoe-/- mice leads to the production of AAAs. Only male mice were studied because female mice have a low incidence of Ang II-induced AAA. 8-10 weeks old male SDC4 -/-apoe-/- mice and Apoe-/- mice were subcutaneously implanted with an osmotic pump (Alzet, MeDOl 2004), which can release AngII at a speed of 1000ng/kg•min for 4 weeks. The mice were fed with a high-fat diet for 4 weeks. Furthermore, SDC4-/-apoe-/- and Apoe-/- mice were subcutaneously implanted with normal saline osmotic pump as control. All mice were fed a high-fat diet for 4 weeks. After the modeling was completed, the mice were sacrificed to evaluate the indicators of abdominal aortic aneurysm in each group, which include the AAA incidence, the survival rate of mice, and the maximum diameter of the abdominal aorta. Elastin van Gieson staining was used to observe the distribution of elastic fibers. The level of MMP2, MMP9, α-SMA, Calponin1, and SM-MHC were observed by western bolt and the level of ROS was detected by DHE staining.
2. CaCl_2_-induced model 8-10 weeks old male SDC4-/- and C57BL/6 mice were anesthetized with isoflurane (1.5-2%), then a midline incision was made in the abdomen to expose the renal artery of the mice. A Gauze soaked with 0.5mol/L CaCl_2_ was directly placed on the inferior segment of the abdominal aorta of the mice for 15 minutes. In control mice, cotton gauze with NaCl (0.9%) was used for the sham operation. All mice were fed a normal diet. After 4 weeks, the mice were sacrificed to evaluate the AAA-related indicators in each group.

**Histological analysis**

After 4 weeks of modeling, the mice were sacrificed, the blood was washed clean by perfusion of the left ventricle with normal saline, and the vessels from the aortic arch to the iliac artery segment were dissected and fixed in 4% formalin. The abdominal aortic segment was gradually dehydrated and embedded in paraffin, and thick sections of 5um were prepared. Human abdominal aortic aneurysm tissues and adjacent tissues were also fixed in 4% formalin to prepare paraffin sections 5um thick.

For H.E. staining, hematoxylin and eosin staining were used to analyze the gross morphology of vessels and lumen diameter.

For Immunofluorescence staining, the deparaffinized tissue slides were boiled in citrate buffer solution at 100℃ for 1 hour to extract the antigen, then were blocked in 1% fetal bovine serum at room temperature for 1 hour, and then incubated with primary antibody at 4℃ overnight. Washing with PBS for 2-3 times, 5 minutes each time, then incubate the secondary antibody at room temperature for 1 hour, wash with PBS for 2-3 times, DAPI staining, apply at room temperature for 10min, wash with PBS twice in the absence of light. The anti-fluorescence quencher was blocked and photographed by a confocal microscope（Leica,TCSSP8, objective 200x）immediately.

For elastic fiber staining, Paraffin sections were stained with a commercial kit (Solarbio, G1593) according to the manufacturer’s instructions to observe the degradation of the elastic fiber. a standard score was used for the semi-quantification of elastin degradation: score 1, no degradation; score 2, mild degradation; score 3, severe degradation; score 4, aortic rupture.

**Immunofluorescence staining report for ROS detection (Frozen-slides)**


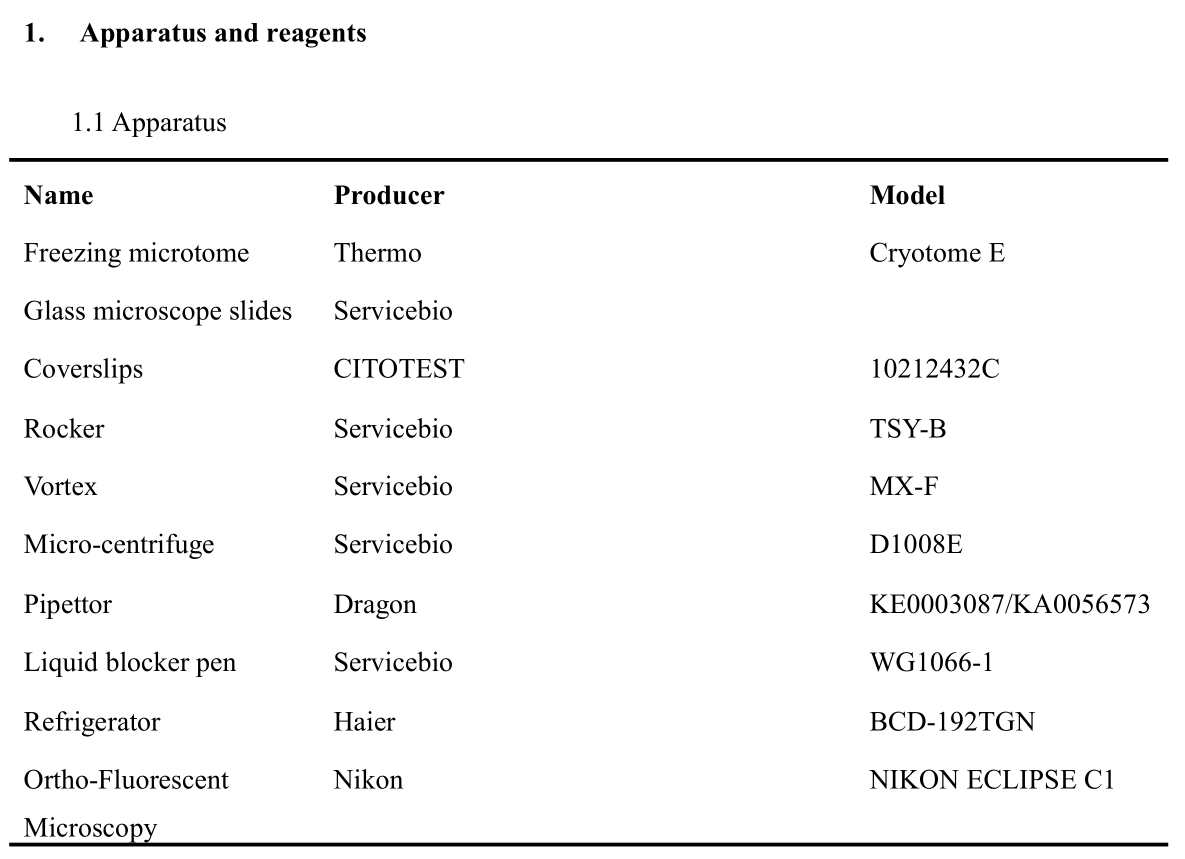


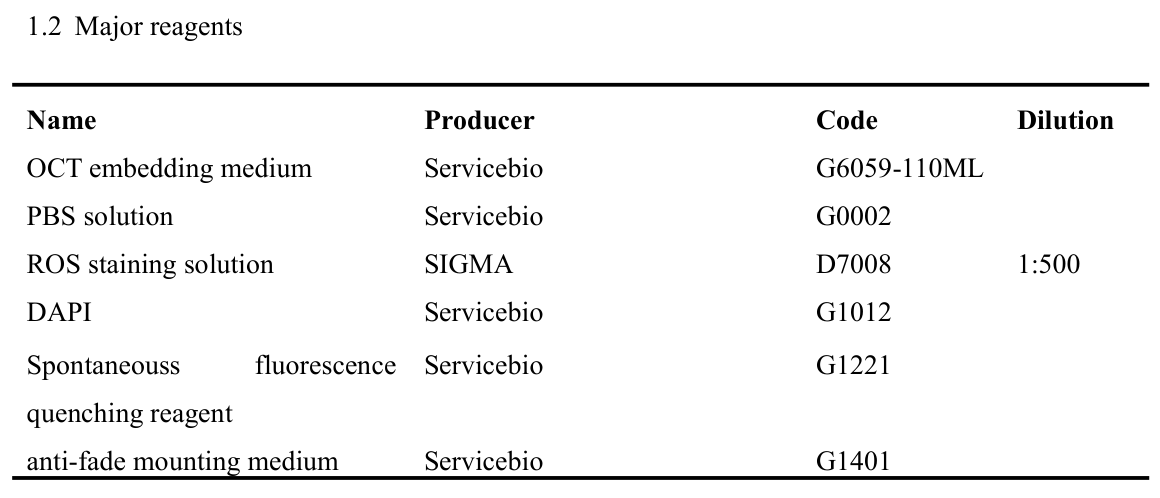


**2. Procedure**

2.1 Circle and Spontaneous fluorescence quenching: restore frozen slides to room temperature.

eliminate obvious liquid, mark the objective tissue with a liquid blocker pen. Add spontaneous

fluorescence quenching reagent to incubate for 5 min. Wash in running tap water for 10 min.

2.2 Staining： add ROS staining solution to the marked area, incubate at 37℃ for 30 min kept in a dark place.

2.3 DAPI counterstain in the nucleus: wash three times with PBS (pH 7.4) in a Rocker device, 5 min each. Then incubate with DAPI solution at room temperature for 10 min, kept in dark place.

2.4 Mount: wash three times with PBS (pH 7.4) in a Rocker device, 5 min each. Throw away

liquid slightly, then coverslip with anti-fade mounting medium.

2.5 Microscopy detection and collect images by Fluorescent Microscopy. DAPI glows blue by

UV excitation wavelength 330-380 nm and emission wavelength 420 nm; CY3 glows red by excitation wavelength 510-560 nm and emission wavelength 590 nm.

**3 Results**

The nucleus is blue by labeling with DAPI. ROS positive cells labeled by fluorescein are red.

**Cell culture and treatment**

Mouse aortic smooth muscle cells (SMCs) were cultured and reproduced in Dulbecco's Modified Eagle's medium (Dulbecco's Modified Eagle's medium (100 g/mL) with 10% fetal bovine serum, streptomycin (100 U/mL), and penicillin (100 U/mL), purchased from Beinart Biotech. The cells were grown for 3 ~ 6 generations and reached 70% ~ 80% confluency for the experiment. Next, RNA (siRNA)-SDC4-knockdown, LV-SDC4 -overexpression, and RNA (siRNA)-vector lentivirus were added into the cell culture medium at MOI=100 for cell transfection. 24 hours later, the culture medium was replaced with a normal medium, and 72 hours after infection, the infection efficiency was detected, and subsequent experiments were conducted. During the process, the cell medium could be changed to maintain the cell activity. After transfection, AngII (1umol/L) was added for 24 hours for subsequent experiments.

**Recombinant lentivirus vector assembly and transduction into VSMCs**

In the present study, we first constructed a GV248 vector (hU6-MCS-Ubiquitin-EGFP-IRES-puromycin) carrying Target Seq agTTACGACTTGGGCAAGAAA and was co-transfected with lentivirus backbone plasmid into 293T cells to produce the recombinant lentivirus vector SDC4-RNAi. Another GV248vector carrying control sequence TTCTCCGAACGTGTCACGT was used to generate a negative virus (negative control). Also, we constructed a GV358 vector (Ubi-MCS-3FLAG-SV40-EGFP-IRES-puromycin) carrying syndecan-4 (SDC4) and was co-transfected with lentivirus backbone plasmid into 293T cells to produce the recombinant lentivirus vector LV-SDC4. We generated empty viruses with another GV358 vector without SDC4 cDNA as controls. VSMCs were cultured overnight in the six-well plate at the density of 3-5 × 10^4^ cells/ml. Lentivirus (1 × 10 ~ 9 TU/ml) were diluted with 1ml complete medium containing HitransG P (1 mol / ml, GeneChem, China)) and added to VSMCs. After transfection at 37 ℃ for 24 hours, the virus culture medium was removed, washed 2-3 times with PBS, and replaced with the fresh virus-free medium. After continuous culture for 72 hours, GFP-positive cells were identified as successfully transfected cells. Next, puromycin (5ug/ml) was used to kill the negative cells, and the successfully transfected cells will survive. The SDC4 expression levels were measured by quantitative reverse transcriptase-polymerase chain reaction (qRT-PCR) and western blot.

**Cell Immunofluorescence analysis**

The cells were fixed with paraformaldehyde for 10-15min, then washed with DPBS 2-3 times. Incubated with 0.1% triton-100 for 5min and washed with DPBS 2-3 times. Followed, the cells were blocked with 5%BSA at room temperature for 40 minutes and incubated against the desired antibodies (1;100-1:200) listed in Supplemental Table 3 overnight at 4 °C. Subsequently, The cells were cleaned with PBS and then incubated with a secondary antibody (1:10000) at room temperature in dark for 60min. DAPI was used to stain the nucleus for 10 minutes at room temperature. Finally, the cells were washed with PBS and photographed quickly with confocal microscopy (Leica, TCSSP8). We used the ImageJ software to analyze the fluorescence intensity of cells.

**RNA isolation and quantitative real-time PCR (qRT-PCR)**

The total RNA of each group was extracted with the TRIzol Reagent Invitrogen kit according to the instructions. cDNA was reverse transcribed from 1 μg of total RNA using Transcriptor First Strand cDNA Synthesis Kit (Roche LifeScience). Quantitative real-time PCR (qRT-PCR) was performed with the SYBR Green Reagents Kit (Roche LifeScience). The mRNAs levels were normalized to glyceraldehyde 3-phosphate dehydrogenase (GAPDH). Changes in expression were calculated using the ∆∆Ct method. Data were shown as the mean ± SD from at least three repeated experiments.  The primers used are listed in Table2.

**Western blot analysis**

Protein was extracted from cells or tissues using RIPA buﬀer with protease inhibitors (Sigma). 5× protein loading buffer was added into the sample according to the volume of protein: buffer = 4:1, and then heated them in a metal bath at 60℃ for 10 minutes. Protein electrophoresis was performed using the TGX FastCast acrylamide kit (BIO), then transferred to PVDF membrane (Millipore, Bedford, MA, America). After blocking in 5% skim milk for 2h, the membrane was incubated with primary antibody (1:1000 or 1:500) in a refrigerator at 4℃ overnight. GAPDH was used as the loading control. Next, the membrane was washed in (0.1%) (TBS-T) at least three times for 10 min each and incubated with horseradish peroxidation-enhanced secondary antibody (1:10,000) for 2h. ECL Prime (GE) was used for testing Protein bands according to the manufacturer's instructions. The nuclear protein was detected with PCNA as a loading control and the cytosolic protein was detected with GAPDH as a loading control. The Nuclear and Cytoplasmic Protein Extraction Kit(KGP1100) was used to separate cytosolic MRTF-A and nuclear MRTF-A protein. The blots were stripped and re-probed for the GAPDH immunodetection. The antibodies used in the experiment are shown in Table 3.

**F-actin and G-actin staining**

Grow adherent cells on the surface of a coverslip. Wash cells with PBS to remove the excess medium. Fix cells in 3.7% formaldehyde in PBS at room temperature for 10-15 minutes. Methanol disrupts the cytoskeletal structure or dye binding, which results in the absence of filament staining. Wash three times with PBS. Permeabilize cells in cold 100% acetone at-20℃for 5 minutes. Permeabilization can also be achieved by incubation with 0.1% Triton X-100 in PBS for 5 minutes. Air-dry the samples or immediately rehydrate in PBS for 5-10 minutes. Wash three times with PBS. To the coverslip, add 200 uL of a 9 ug/mL (or 0.3 uM) solution of fluorescent DNase I (Alexa Fluor 594 DNase I conjugates, Invitrogen) in the buffer. Optional: To simultaneously label F-actin, also add I unit (200 L of a 0.165 uM solution) of a fluorescent phallotoxin (Alexa Fluor 647phalloidin, Invitrogen). Stain for 15-20 minutes. Wash three times with PBS. The nuclei were stained with DAPI for 10 minutes and photographed with confocal microscopy (Leica, TCSSP8, objective 200x).

**G-actin / F-actin Assay (**Cytoskeleton, Cat #BK037**)**

1. a) Aspirate media from the dish. Incline dish to 30° angle to help remove as much

media as possible.

b) Add appropriate volume of warm LAS2.

c) Harvest cells by scraping thoroughly with a cell scraper, again keep the plate at a

30° angle to help collect all of the lysates.

2. Homogenize samples using a small handheld or motorized homogenizer suitable for

low milliliter volumes or a 200 µl pipet tip (usually sufficient for cell culture samples).

3. Incubate lysates at 37°C for 10 minutes.

4. Remove 100 µl volume from each lysate for further analysis. Any remaining lysate

can be discarded or used for other purposes at this point.

NOTE: If larger volumes of lysate are required, due to limitations of centrifuge

equipment, make sure that the pellet is resuspended in a volume of F-actin

depolymerization buffer that is equal to the centrifuged lysate volume, i.e. if you

centrifuge 1 ml of lysate then pellets should be resuspended in 1 ml of F-actin

depolymerization buffer.

5. Centrifuge the 100 µl volumes of lysates at 350 x g (approx. 2,000 rpm in a tabletop

microfuge), room temperature for 5 minutes to pellet unbroken cells of tissue debris.

6. Pipette supernatants into clearly labeled ultracentrifuge tubes.

7. Centrifuge at 100,000 x g, 37°C for 1h. This step will pellet F-actin and leave G-

actin in the supernatant.

8. Remove supernatants to fresh tubes designated as supernatant samples.

Supernatants should be removed gently to avoid disturbing the F-actin pellet.

9. Add 100 µl of F-actin depolymerization buffer to each pellet and incubate on ice for

1h to allow actin depolymerization to occur. Pipette up and down several times

every 15 minutes to help pellet resuspension.

10. Add 25 µl of 5X SDS sample buffer to each of the pellet and supernatant samples

and mix well.

11. The samples are now ready for actin quantitation by SDS-PAGE and western blot

analysis. Samples can be stored at –20°C before moving to Actin quantitation by SDS-PAGE / Western blot analysis.

**RhoA detecting**

VSMCs were divided into control, SDC4-KO, and SDC4-OE group, then a commercial Kit (Rho Activation Assay Biochem Kit™, Cat. # BK036-S) was used to assess the proportion of activated RhoA.

1. Run the test protein samples and controls on a 12% SDS gel until the dye

front reaches the bottom of the gel.

2. Equilibrate the gel in Western blot buffer (See recipe below) for 15 min at room

temperature before electro-blotting.

3. Transfer the protein to a PVDF membrane for 45 minutes at 75V.

4. Wash the membrane once with TBS (10 mM Tris-HCl pH 8.0, 150 mM NaCl).

5. Allow the membrane to air dry for 20-30 minutes or overnight at room temperature.

6. Rehydrate the PVDF membrane by soaking it for 3 minutes in methanol. Transfer the

membrane to TBST (10 mM Tris-HCl pH 8.0, 150 mM NaCl, 0.05% Tween 20) at

room temperature for 5 minutes to equilibrate the membrane.

7. Block the membrane surface with 5% nonfat dry milk in TBST for 30 min at room

temperature with constant agitation.

8. Incubate the membrane with a 1:500 dilution of anti-RhoA antibody (Cat. # ARH05,

provided with the kit) diluted in TBST (no blocking agent) for 2-3 h at room temperature

or overnight at 4°C with constant agitation.

9. Rinse the membrane in 50 ml TBST for 1 min.

10. Incubate the membrane with an appropriate dilution (eg. 1:20,000) of anti-mouse

a secondary antibody that can recognize mouse IgM (eg. goat anti-mouse HRP

conjugated IgG from Jackson Labs., Cat. # 115-035-068) in TBST for 30 min-1 h at

room temperature with constant agitation.

11. Wash the membrane 5 times in TBST for 10 min each.

12. Use an enhanced chemiluminescence detection method to detect the RhoA signal.

**Enzyme-linked immunosorbent assay (ELISA)**

The supernatant of control, SDC4-KO, and SDC4-OE cells, and the supernatant of control, SDC4-KO, and SDC4-OE cells after AngII treatment for 24 h were extracted respectively. The expressions of MMP2, TNF-α, IL-1, and IL-6 were detected with a specific ELISA kit (MultiSciences).
