# Supplementary figures and images for: A reduction in the vascular smooth muscle cell focal adhesion component syndecan‐4 is associated with abdominal aortic aneurysm formation
Source: Clin Transl Med. 2021 Dec 22;11(12):e605. doi: 10.1002/ctm2.605 (PMC8693440; doi:10.1002/ctm2.605)

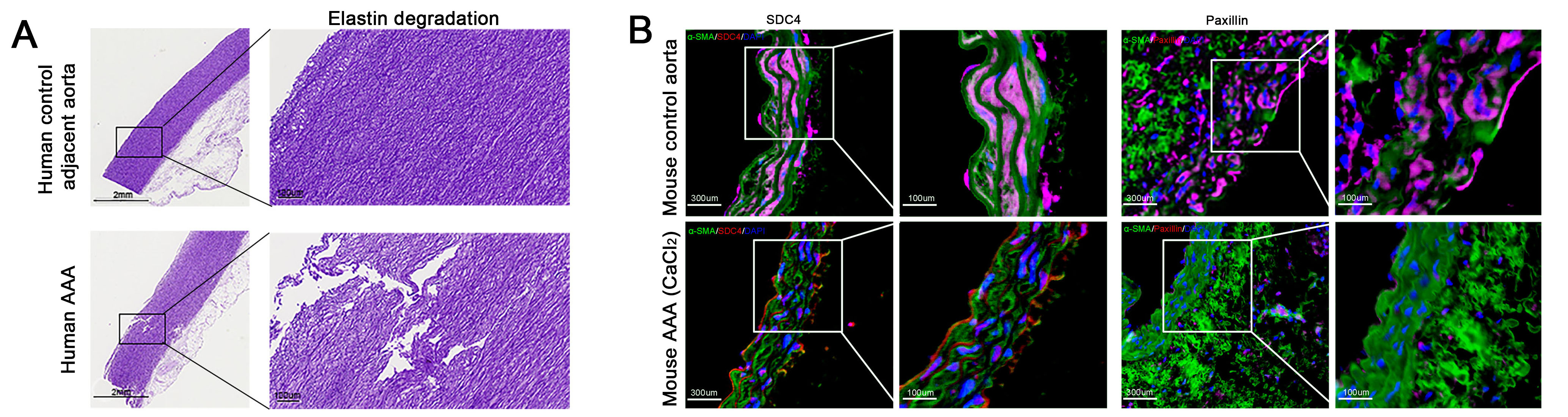

Supplement: Supplementary file 2 — Supporting Information [file CTM2-11-e605-s004.jpg]

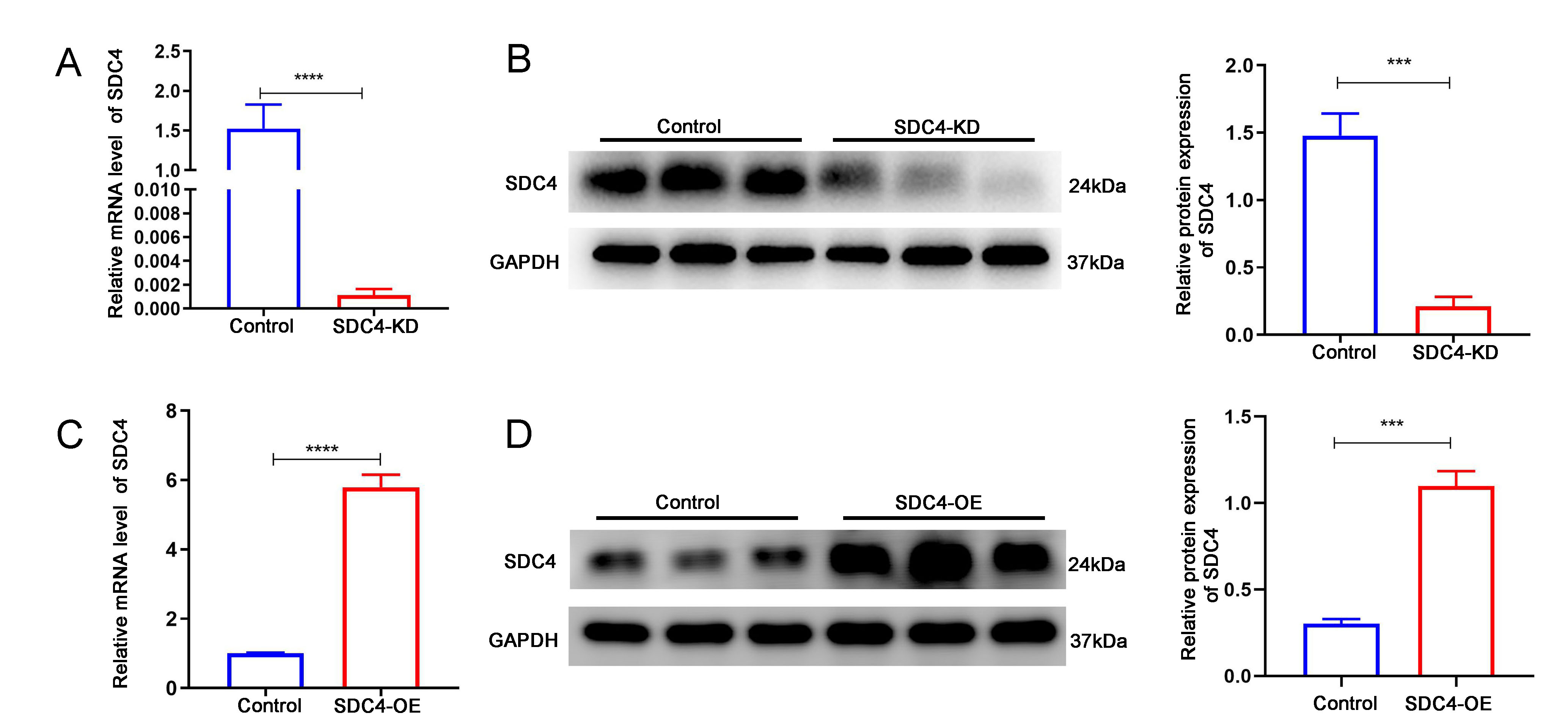

Supplement: Supplementary file 3 — Supporting Information [file CTM2-11-e605-s006.jpg]

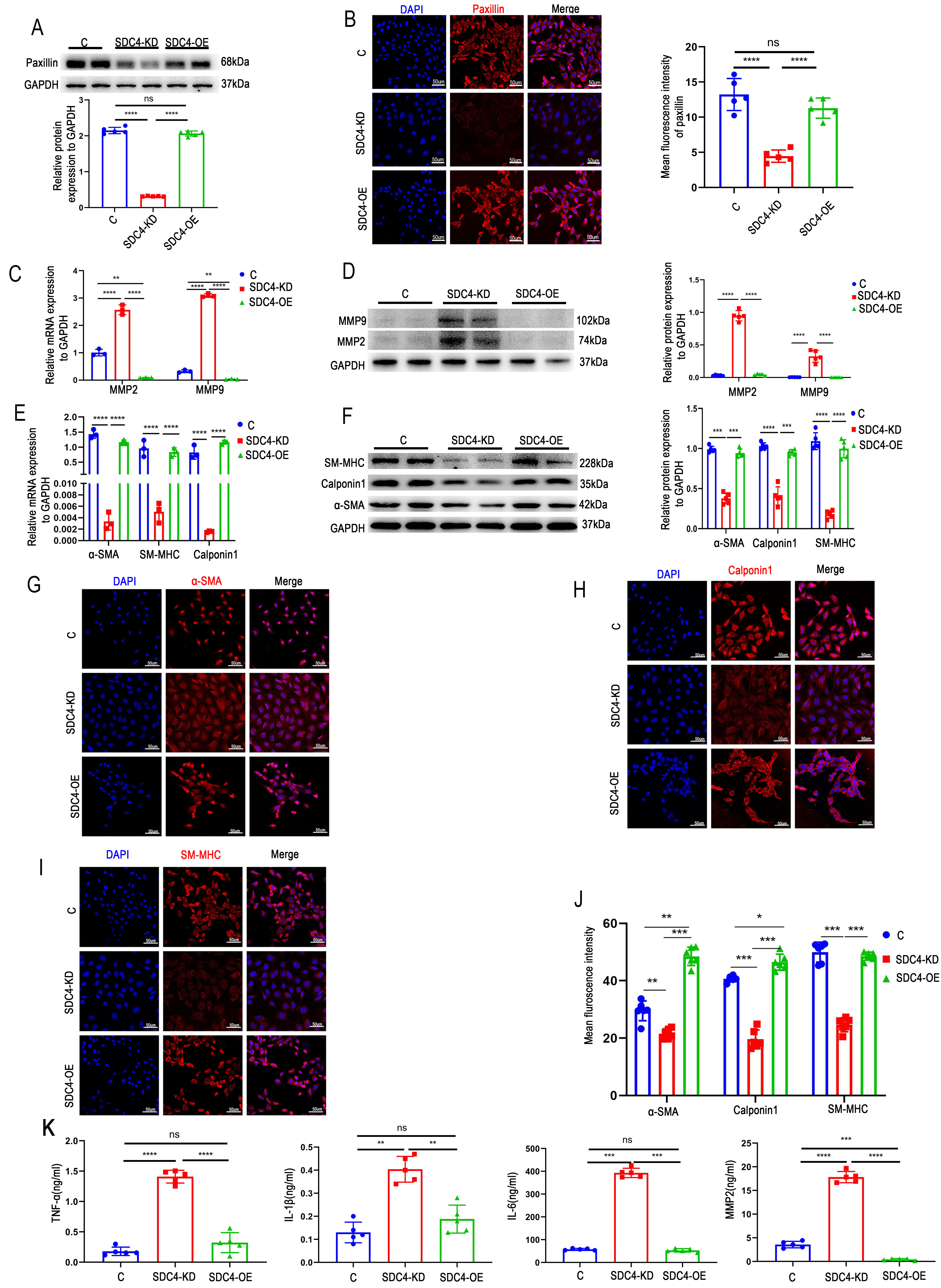

Supplement: Supplementary file 4 — Supporting Information [file CTM2-11-e605-s002.jpg]

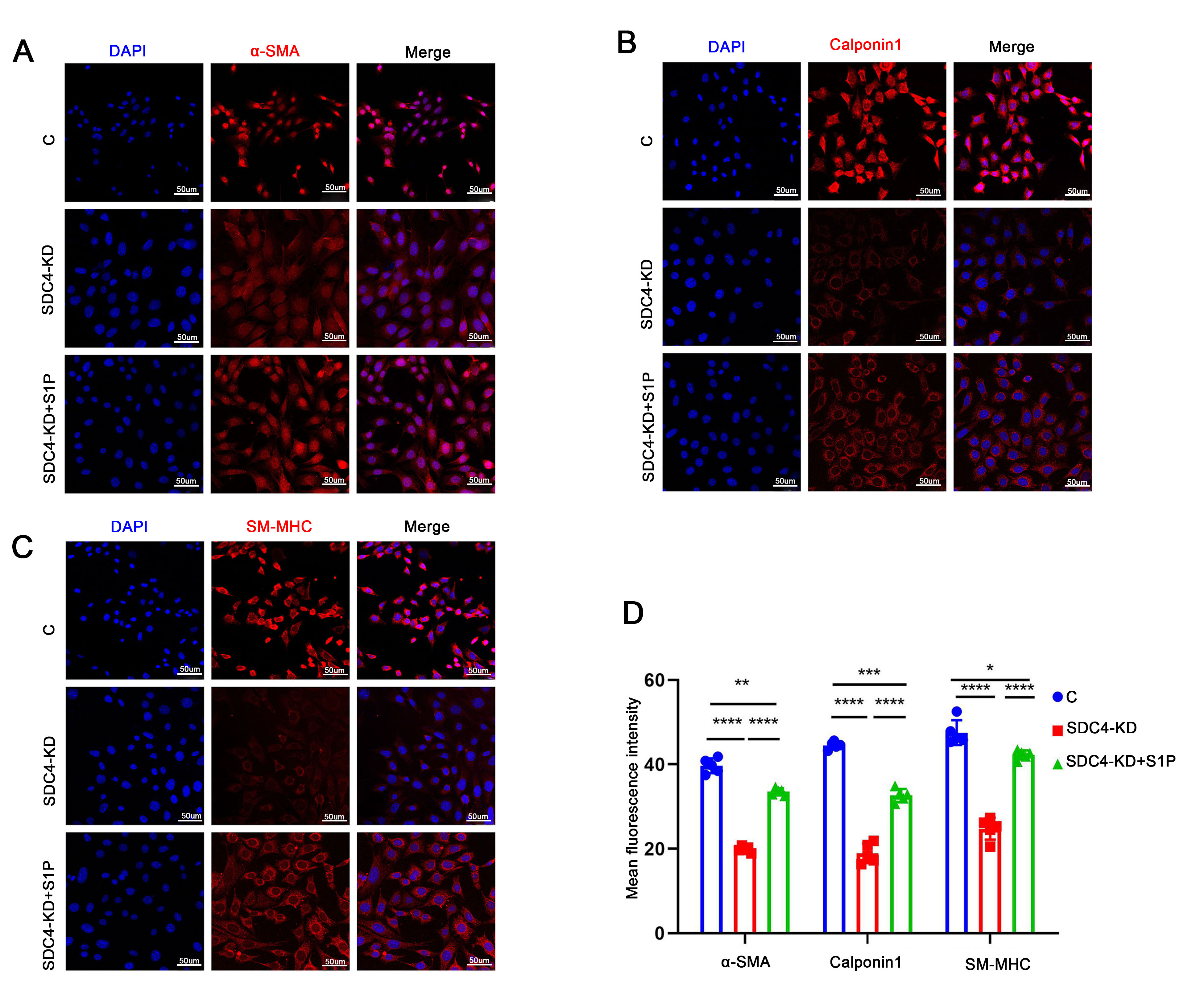

Supplement: Supplementary file 5 — Supporting Information [file CTM2-11-e605-s003.jpg]

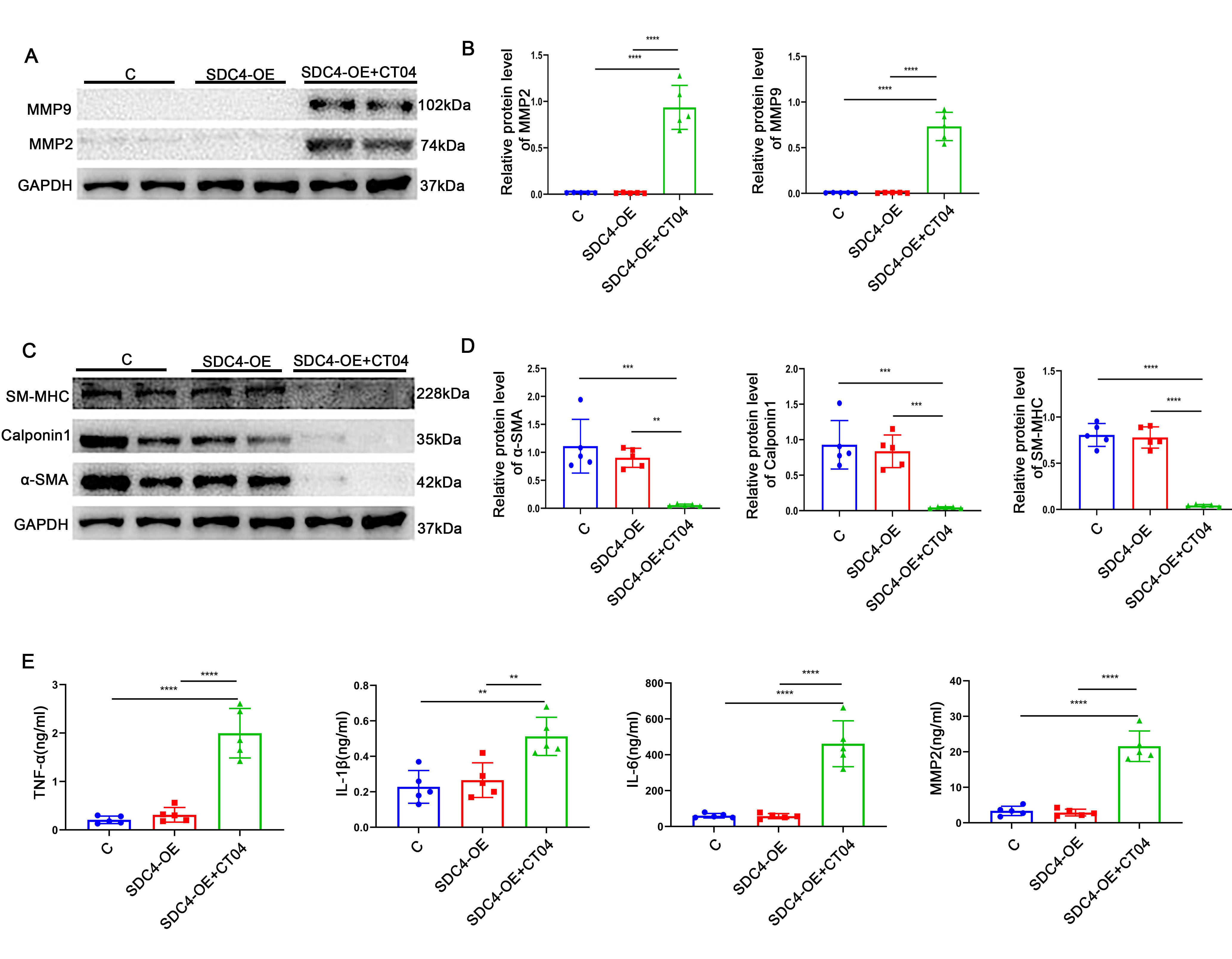

Supplement: Supplementary file 6 — Supporting Information [file CTM2-11-e605-s007.jpg]

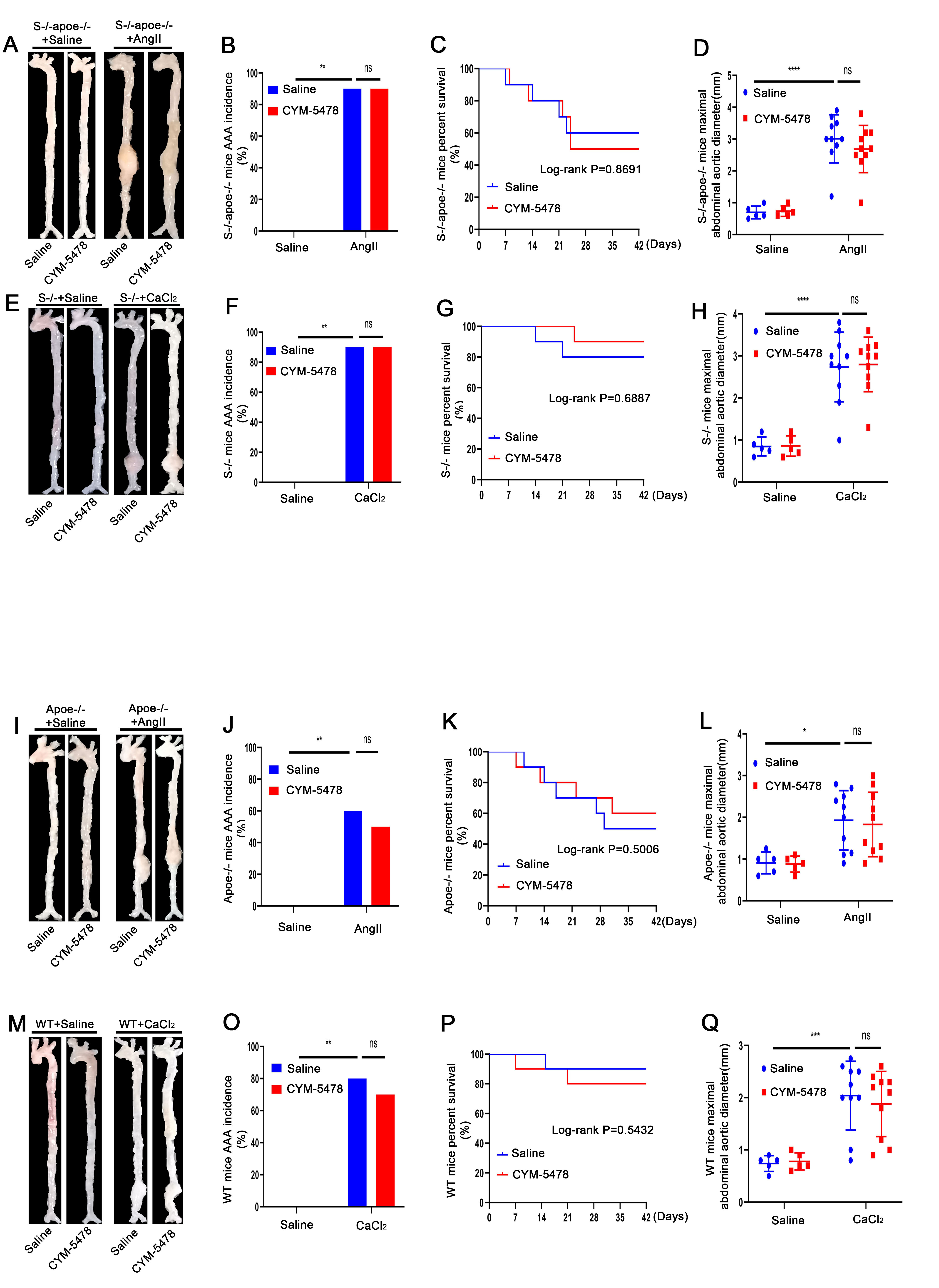

Supplement: Supplementary file 7 — Supporting Information [file CTM2-11-e605-s005.jpg]
